# Intragenic complementation at the *Lotus japonicus CELLULOSE SYNTHASE-LIKE D1* locus rescues root hair defects

**DOI:** 10.1093/plphys/kiab204

**Published:** 2021-05-06

**Authors:** Bogumil J Karas, Loretta Ross, Mara Novero, Lisa Amyot, Arina Shrestha, Sayaka Inada, Michiharu Nakano, Tatsuya Sakai, Dario Bonetta, Sushei Sato, Jeremy D Murray, Paola Bonfante, Krzysztof Szczyglowski

**Affiliations:** 1 Department of Biochemistry, The University of Western Ontario, London, Ontario, Canada, N6A 5C1; 2 Agriculture and Agri-Food Canada, London Research and Development Centre, London, Ontario, Canada, N5V 4T3; 3 Department of Life Sciences and Systems Biology, University of Torino, Torino, Italy; 4 RIKEN Plant Science Center, 1-7-22 Suehiro-cho, Tsurumi-ku, Yokohama, Kanagawa 230-0045, Japan; 5 Graduate School of Science and Technology, Niigata University, 8050 Ikarashi-nino-cho, Nishiku, Niigata 950-2181, Japan; 6 Faculty of Science, Ontario Tech University, Oshawa, Ontario, Canada; 7 Graduate School of Life Sciences, Tohoku University, 2-1-1 Katahira, Aoba-ku, Sendai, 980-8577, Japan; 8 National Key Laboratory of Plant Molecular Genetics, CAS-JIC Centre of Excellence for Plant and Microbial Science (CEPAMS), CAS Center for Excellence in Molecular and Plant Sciences, Institute of Plant Physiology and Ecology, Chinese Academy of Sciences, Shanghai, 200032, China; 9 Department of Biology, University of Western Ontario, London, Ontario, N6A 5B7 Canada

## Abstract

Root hair cells form the primary interface of plants with the soil environment, playing key roles in nutrient uptake and plant defense. In legumes, they are typically the first cells to become infected by nitrogen-fixing soil bacteria during root nodule symbiosis. Here, we report a role for the *CELLULOSE SYNTHASE-LIKE D1* (*CSLD1*) gene in root hair development in the legume species *Lotus japonicus*. CSLD1 belongs to the cellulose synthase protein family that includes cellulose synthases and cellulose synthase-like proteins, the latter thought to be involved in the biosynthesis of hemicellulose. We describe 11 *Ljcsld1* mutant alleles that impose either short (*Ljcsld1-1*) or variable (*Ljcsld1-2* to *11*) root hair length phenotypes. Examination of *Ljcsld1-1* and one variable-length root hair mutant, *Ljcsld1-6*, revealed increased root hair cell wall thickness, which in *Ljcsld1-1* was significantly more pronounced and also associated with a strong defect in root nodule symbiosis. *Lotus japonicus* plants heterozygous for *Ljcsld1-1* exhibited intermediate root hair lengths, suggesting incomplete dominance. Intragenic complementation was observed between alleles with mutations in different CSLD1 domains, suggesting CSLD1 function is modular and that the protein may operate as a homodimer or multimer during root hair development.

## Teaser

Intragenic complementation reveals that *Lotus japonicus CELLULOSE SYNTHASE-LIKE D1* multimers facilitate root hair development.

## Introduction

Plant cell walls constitute a dynamic yet rigid interface between cells or between the cell and the external environment. While providing structural support and protection, they also act together with cell membranes as important filtering structures. Composed primarily of cellulose, hemicellulose, and pectin (Lampugnani et al., 2018), cell walls vary considerably based on plant species as well as tissue type (Popper et al., 2014; Höfte and Voxeur, 2017; Penning et al., 2019). Nonetheless, they share an important feature in their propensity for rapid remodeling both during isotropic and anisotropic expansions; this remodeling is a key to growth and survival of cells and organisms, including their ability to respond to various abiotic and biotic cues.

Most research aimed at identifying the various players required for cell wall synthesis and remodeling has been performed in the model plant Arabidopsis (*Arabidopsis thaliana*) (Liepman et al., 2010). Cellulose, the main structural component of plant cell walls, is synthesized by large, plasma membrane-localized complexes consisting of cellulose synthase (CESA) subunits. In Arabidopsis, a family of 10 genes encodes CESA proteins (Richmond and Somerville, 2000). CESA1-, CESA3-, and CESA6-related proteins (CESA2/5/6/9) mediate cellulose biosynthesis in the primary cell wall, while CESA4, CESA7, and CESA8 are involved in secondary cell wall formation (Arioli et al., 1998; Taylor et al., 2003; Desprez et al., 2007; Persson et al., 2007; Lampugnani et al., 2019).

Arabidopsis also has 30 *CELLULOSE SYNTHASE-LIKE* (*CSL*) genes, and their protein products have been classified into six groups: CSLA, CSLB, CSLC, CSLD, CSLE, and CSLG. Two additional groups, CSLF and CSLH, have been identified in grasses (Lerouxel et al., 2006) and a third group, CSLJ, is present in grasses and also some dicots (Yin et al., 2009). Compared with CESAs, the functional relevance of CSLs is less well understood and based on their diverse expression patterns, they are thought to play more specialized roles. Several studies have indicated that CSLs are involved in the synthesis of hemicellulose, a group of heterogeneous polysaccharides, such as xylan, glucomannan, and xyloglucan, which provide additional structural support to the cell wall through their interactions with cellulose. For instance, Arabidopsis CSLAs were shown to have mannan and glucomannan synthase activity (Liepman et al., 2005; Goubet et al., 2009). Heterologously expressed CSLD2, CSLD3, and CSLD5 also showed mannan synthase activity (Yin et al., 2011), and CSLCs were implicated in xyloglucan backbone synthesis. Interestingly, chimeric CSLD3, containing the CESA6 catalytic domain, could complement the *csld3* mutant phenotype (Park et al., 2011), indicating some level of functional overlap between CSLD and CESA proteins. Consistent with this observation, more recent studies demonstrated that cotton (*Gossypium hirsutum*) CSLD3 could restore cell elongation and wall integrity in an Arabidopsis *cesa6* mutant (Hu et al., 2019) and that Arabidopsis CSLD3 is a β-1,4-glucan synthase (Yang et al., 2020).

Among the CSLs, CSLD group members share the highest similarity with CESAs (Richmond and Somerville, 2000; Carroll and Specht, 2011), including a conserved β-glycosyltransferase D, D, D, QXXRW motif located in the active site of the enzymes (Saxena et al., 1995; Richmond and Somerville, 2000). Studies on *csld* mutants have suggested a role for these genes in stem and tip cell growth, as well as cellulose deposition. For example, mutations in *CSLD5* have been associated with defects in stem growth (Bernal et al., 2007), in *CSLD1* and *CSLD4* with pollen tube development (Bernal et al., 2008; Wang et al., 2011), in *CSLD2* and *CSLD3* with defective root hairs (Favery et al., 2001; Wang et al., 2001; Bernal et al., 2008; Galway et al., 2011), or in plants lacking both CSLD2 and CSLD3, with defective female gametophyte development (Yoo et al., 2012).

Root hair cells are large, rapidly growing, and easily observable, making them an ideal system for studying cell wall development (Favery et al., 2001). We previously identified root hair mutants from a screen for genetic suppressors of the *Lotus japonicus hypernodulation aberrant root formation1-1* (*har1-1*) hypernodulation phenotype (Murray et al., 2006a, 2006b), where developmental defects in the root epidermis led to the impairment of root nodule symbioses. Through microscopic observations and genetic crosses, we classified these root hair mutants into four complementation groups: *hairless*, corresponding to the *L. japonicus Root Hairless* locus (*LjRHL*); *petite* (*L. japonicus Petite Root Hairs, LjPRH*); *short* (*L. japonicus Short Root Hairs, LjSRH*); or *variable* (*L. japonicus Variable Root Hairs*, *LjVRH*; Karas et al., 2005). The *LjRHL* locus was identified as a basic helix–loop–helix protein, and its putative orthologs *AtLRL1*, *AtLRL2*, and *AtLRL3* are redundantly required for root hair development in Arabidopsis (Karas et al., 2009).

In this study, we demonstrated through map-based cloning that *L. japonicus* short (*srh*) and variable (*vrh*) root hair mutant lines carried lesions in the *CSLD1* gene, which encodes a protein with the highest sequence similarity to members of the Arabidopsis CSLD family. We show here that the *L. japonicus* CSLD family, like Arabidopsis, has six members. The root hairs of *csld1* mutants had thicker cell walls, which in *Ljcsld1-1* was particularly pronounced and was associated with impaired colonization of roots by *Mesorhizobium loti*, a nitrogen-fixing symbiont of *L. japonicus*. A subset of allelic crosses resulted in the restoration of wild-type like root hairs, reflecting intragenic complementation and thus suggesting that LjCSLD1 forms multimers during root hair development.

## Results

### Identification of 11 *Ljcsld1* mutant alleles

**Figure 1:**
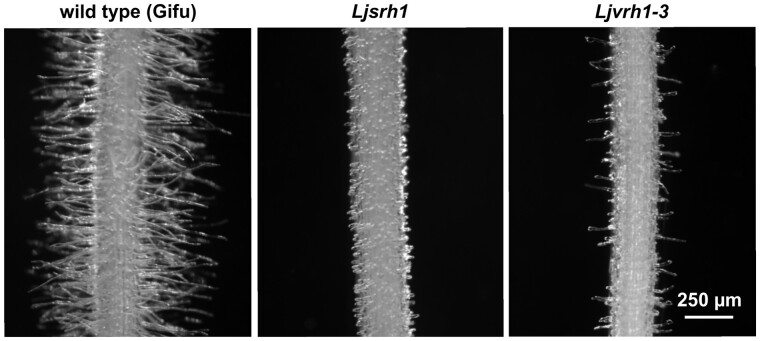
Root hair phenotypes. Representative root segments of wild-type *L. japonicus* Gifu and the *Ljcsld1-1* (short; initially *Ljsrh1*) and *Ljcsld1-2* (variable; initially *Ljvrh1-3*) mutant lines are shown. Images were taken at approximately 0.5–1 cm from the root tip, where fully elongated root hairs are present in Gifu (see Karas et al., 2005). The 250-µm scale applies to all three images.

The identification and phenotypic characterization of three allelic *L. japonicus* mutant lines of the variable root hair phenotype (*Ljvrh1-1*, *Ljvrh1-2*, and *Ljvrh1-3*) and one additional line that showed a short root hair phenotype (*Ljsrh1*) were described earlier (Karas et al., 2005). A survey of our in-house *L. japonicus* mutant collection and the National BioResource Project (NBRP) Legume Base resource (https://www.legumebase.brc.miyazaki-u.ac.jp/) identified 26 additional lines with altered root hair phenotypes. While none of these lines had short root hairs, 15 showed phenotypes resembling *Ljvrh1* (Figure 1).

A map-based cloning approach was employed to identify the underlying genetic lesions. A 0.8 cM genetic interval at the bottom of *L. japonicus* chromosome III was previously defined to contain the *LjVRH1* and *LjSRH1* (Karas et al., 2005). This region, delineated by flanking markers TM1419 and TM0127, encompassed five overlapping TAC clones (Supplemental Figure S1). A survey of genes present on these clones identified, among others, a *Cellulose Synthase-Like D* gene (*LjCSLD1*) located on TM0757 (Supplemental Figure S1). This gene was considered as a viable candidate for either the *LjVRH1* or the *LjSRH1* locus, as proteins belonging to the Arabidopsis AtCSLD subfamily, such as KOJAK, were shown to be required for root hair cell morphogenesis (Favery et al., 2001). Sequencing of the predicted coding region of *LjCSLD1* in wild-type and the three *Ljvrh* mutants identified a C3494T transition in *Ljvrh1-1*, while *Ljvrh1-2* and *Ljvrh1-3* carried C3939T and G530A transitions, respectively. The same locus was amplified and sequenced from the 15 additional *L. japonicus* mutant lines with the *Ljvrh-*like phenotypes. Seven of these carried single-nucleotide substitutions within the predicted coding region of the *LjCSLD1* gene (Supplemental Table S1), while the remaining lines had the wild-type *LjCSLD1* sequence, suggesting that their mutant root hair phenotypes were determined by mutations in an independent locus or loci.

A parallel effort to map-based clone the *LjSRH1* locus continued as the initial genetic complementation analysis yielded F1 plants with wild-type like root hairs, suggesting that *LjSRH1* was independent from *LjVRH1*. The position of the *Ljsrh1* mutation was further delimited to the 50-kb region between flanking markers JM010 and JM003, which contained eight predicted genes, including *LjCSLD1* (Supplemental Figure S1). Sequencing of all eight genes from wild-type and the *Ljsrh1* mutant identified a single-nucleotide substitution, C220T, in *LjCSLD1*, while the nucleotide sequence of the remaining seven genes was wild-type. The *Ljsrh1* allele was tentatively renamed as *Ljcsld1-1*. The same *Ljcsld* nomenclature was used for the variable root hair phenotype-associated mutations, with allele numbering reflective of the relative position of a given mutation along the *LjCSLD1* gene sequence (Supplemental Table S1).

### *Lotus japonicus LjCSLD1* and Arabidopsis *AtCSLD2* and *AtCSLD3* are functionally conserved

**Figure 2:**
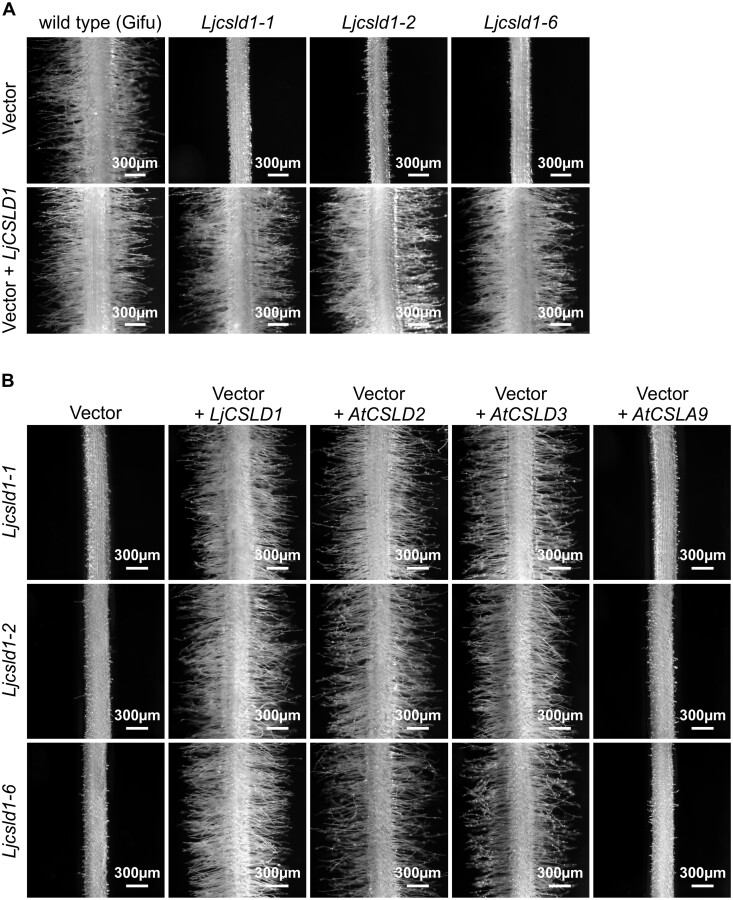
Genetic complementation. A, The *LjCSLD1* gene complements the *Ljcsld1-1* short and *Ljcsld1-2* and *Ljcsld1-6* variable root hair phenotypes. The mutant plants were transformed by either *A. rhizogenes* strain AR10 carrying control binary vector with no insert (vector, upper row), or the same vector with the entire genomic version of the *LjCSLD1* gene (vector + *LjCSLD1;* bottom row). B, Arabidopsis *AtCSLD2* and *AtCSLD3* complement the short root hair phenotype of *Ljcsld1-1* (top row) and the variable root hair phenotypes of *Ljcsld1-2* (middle row) and *Ljcsld1-6* (bottom row) while *AtCSLA9* does not. The corresponding Arabidopsis cDNAs were expressed in transgenic hairy roots under the control of *CaMV* 35S promoter. The empty pEarley101 binary vector, containing the 35S promoter, was used as negative control, while *LjCSLD1* cDNA served as the positive control.

To confirm that mutations at the *LjCSLD1* locus were causative to both short and variable root hair phenotypes*, in planta* complementation experiments were performed. A binary vector containing an 8.5-kb genomic fragment encompassing the entire *LjCSLD1* locus was introduced into roots of *Ljcsld1-1, Ljcsld1-2*, and *Ljcsld1-6* mutant lines by *Agrobacterium rhizogenes*-mediated transformation (Murray et al., 2007). The resulting transgenic hairy roots produced wild-type-like root hairs. In contrast, a control transformation, using an *A. rhizogenes* strain carrying an empty vector, failed to complement the defective root hair phenotypes (Figure 2A).

**Figure 3:**
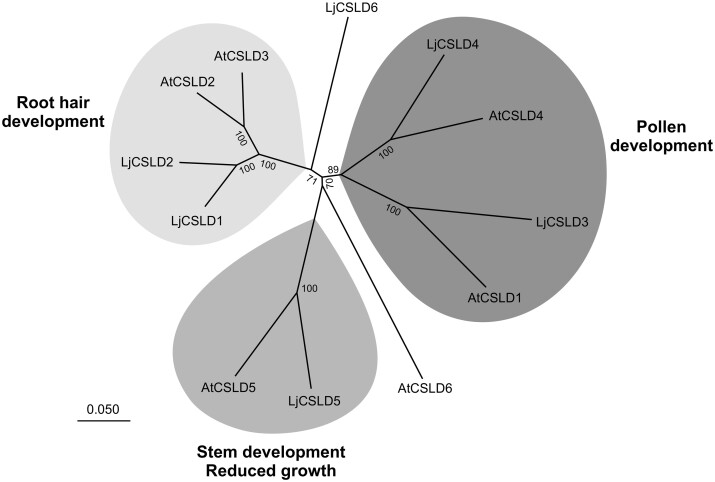
Unrooted relationship tree based on full-length sequences of predicted Cellulose Synthase-Like D proteins of *L*. *japonicus* and *A*. *thaliana*. The scale bar represents the number of amino acid differences between sites (i.e. 0.050 means 5% sequence dissimilarity). Note that in addition to indicated group functions, some of the proteins partake in other developmental processes.

Using bioinformatic analysis we subsequently identified six members of the *L. japonicus CELLULOSE SYNTHASE-LIKE D1 (CSLD)* gene family (Figure 3). Given the amino acid similarity between LjCSLD1 and Arabidopsis AtCSLD2 and AtCSLD3 (Supplemental Table S2), and the involvement of AtCSLD2 and AtCSLD3 in root hair development (Favery et al., 2001; Bernal et al., 2008), an inter-species complementation test was performed. Expression of *AtCSLD2* and *AtCSLD3* under the control of the *CaMV* 35S promoter in transgenic hairy roots rescued the short and variable root hair phenotypes of *L. japonicus Ljcsld1-1*, *Ljcsld1-2*, and *Ljcsld1-6* mutants. In contrast, the more distantly related *AtCSLA9* (AT5G03760.1; Richmond and Somerville, 2000) did not complement the root hair defects of any of the three allelic lines tested (Figure 2B). Based on these data, we concluded that the *LjCSLD1* gene, identified through map-based cloning, indeed corresponded to the *L. japonicus LjVRH1/LjSRH1* locus and is functionally equivalent to the Arabidopsis *AtCSLD2* and *AtCSLD3* genes.

### Structure of the *LjCSLD1* gene and protein

Analysis of the *LjCSLD1* gene sequence (Supplemental File 1) showed that it comprised four exons and three introns, and was predicted to produce an mRNA of 3,805 nt in length with a 3450-nt long open reading frame (ORF) encoding the predicted LjCSLD1 protein of ∼129 kDa. The ORF was flanked by 152 and 203 nt long 5′ and 3′-untranslated regions, respectively (Supplemental Table S3).

**Figure 4:**
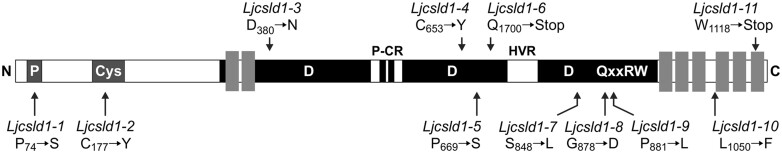
Schematic of the LjCSLD1 protein structure showing positions of the molecular lesions for identified mutant alleles. Black boxes represent conserved sequences among members of the CSLD protein family and gray rectangles represent transmembrane domains; (P), PPTP region; (Cys), cysteine-rich region; (P-CR), the plant-conserved region; (HVR), plant hyper-variable region; D, D, D, QxxRW represents a conserved β-glycosyltransferase motif where D is aspartic acid; Q, glutamine; x, variable residue; R, arginine and W, tryptophan.

The LjCSLD1 protein was predicted to have two cysteine (C)-rich motifs of CX_4_CX_15_CXC and CX_2_CX_11_CX_2_C, which are mostly conserved with AtCESA1 (Supplemental Figure S2, A and B). Furthermore, four sub-domains (U1–U4), encompassing three highly conserved aspartic acid residues (D) and the QXXRW motif that are characteristic of the processive β-glucosyltransferases in plants and bacteria, were present in the presumed globular region of LjCSLD1 (Supplemental Figure S2A). This region was flanked by eight predicted transmembrane segments; two of these segments were present in the N-terminal portion of the region, while the remaining six transmembrane domains were located in the C-terminal region (Figure 4; Supplemental Figure S2A).

Two mutations, *Ljcsld1-1* and *Ljcsld1-2*, affected the LjCSLD1 N-terminal region, where substitutions of P_74_ to S and C_177_ to Y have occurred, respectively (Figure 4; Supplemental Table S1). P_74_ resides within a short proline-rich region (PPTP) located close to the N-terminus of LjCSLD1 (Supplemental Figure S2A). This proline-rich region is conserved in CSLD proteins (Supplemental Figure S3) but absent from cellulose synthase catalytic subunit proteins (CESA) and other members of the CSL protein family. The C_177_ residue constitutes a part of the LjCSLD1 C-rich region. Seven additional mutations, *Ljcsld1-3* to *Ljcsld1-9*, mapped to the predicted globular region of the LjCSLD1 protein. With the exception of *Ljcsld1-6*, where a single-nucleotide change of C3494T resulted in a premature stop codon, all remaining mutations in this region were nonsynonymous, leading to amino acid substitutions. Given the high level of amino acid sequence conservation (Supplemental Figure S3), it was not surprising to find that all of these mutations altered amino acid residues that are conserved in CSLD proteins. Notably, however, two of these mutations, *Ljcsld1-7* and *Ljcsld1-9*, were located within the highly conserved U3 and U4 domains of LjCSLD1 (Supplemental Figure S2A). The two remaining mutations, *Ljcsld1-10* and *Ljcsld1-11*, affected the C-terminal portion of the protein, encompassing six transmembrane domains (Figure 4; Supplemental Table S1). The *Ljcsld1-10* mutation resulted in a nonsynonymous change, leading to an amino acid substitution, while *Ljcsld1-11* caused a premature stop codon. *Ljcsld1-6*, and perhaps also *Ljcsld1-11*, could represent null alleles.

### Intra-allelic complementation at the *LjCSLD1* locus

**Figure 5:**
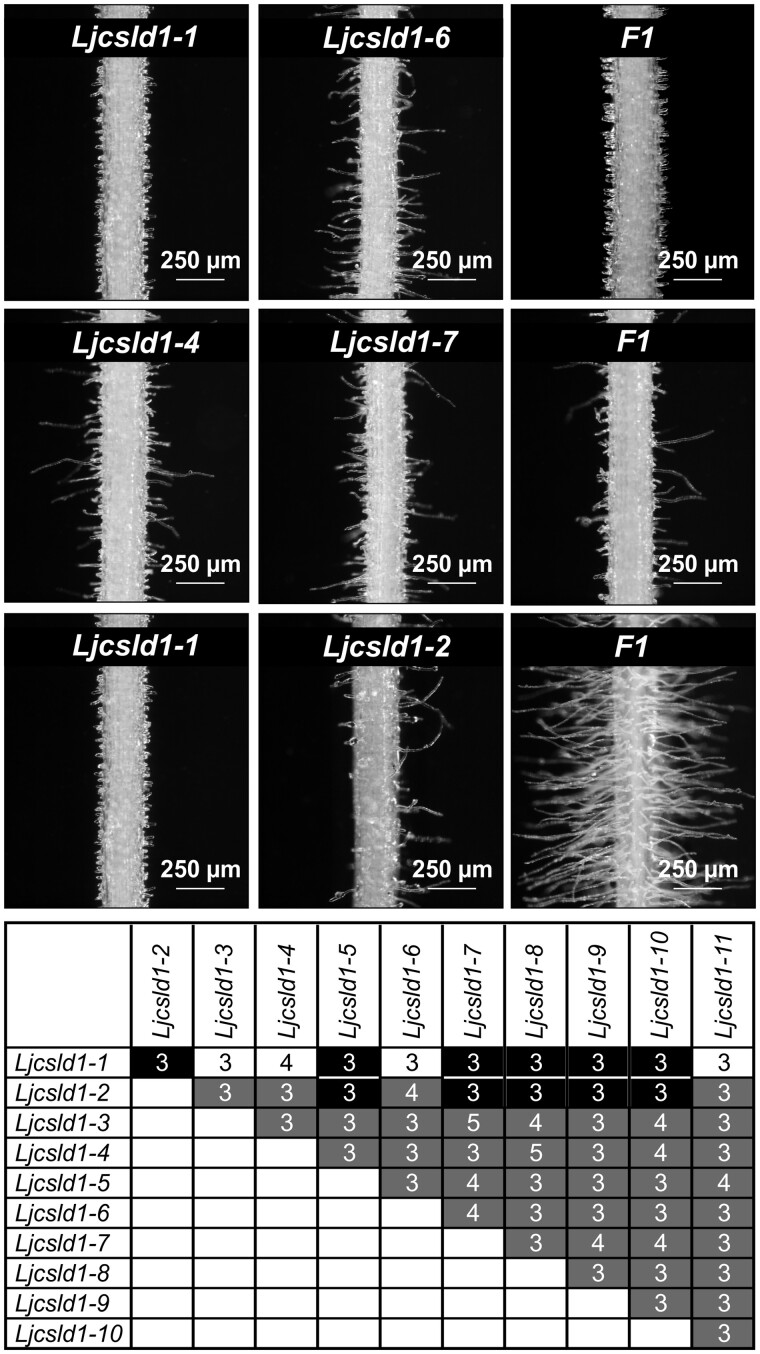
Intragenic complementation at the *LjCSLD1* locus. Allelic crosses were performed among all 11 *Ljcsld1* variants. Representative examples of three different root hair phenotypes, short (top row), variable (middle row), and wild-type (bottom row), as recovered in F1, are shown along with phenotypes of parental lines. Root segments were photographed at approximately 0.5–1 cm from the root tip. The table summarizes results of all allelic crosses; shaded boxes represent one of the three F1 root hair phenotypes: black—wild type-like, white—short, gray—variable root hairs. The numbers of independent crosses for each allelic combination are indicated in the shaded boxes. For each cross, two to five F1 plants were analyzed—and in all cases they showed the same root hair phenotype.

Our initial expectation that two independent loci were involved in the observed variable and short root hair phenotypes was based on the observation that the cross between homozygous *Ljcsld1-1* (short root hair phenotype) and *Ljcsld1-2* (variable root hair phenotype) yielded F1 progeny with a wild-type-like root hair phenotype (Karas et al., 2005)*.* As mutations at a single locus, *LjCSLD1*, were found to be causative for both phenotypes, this suggested that intra-allelic complementation must have accounted for the observed phenotypic rescue effect. We further tested this assumption by analyzing progeny from genetic crosses encompassing all 11 *Ljcsld1* alleles (Figure 5).

As expected for crosses between different alleles of a single gene, the majority resulted in F1 progeny with short or variable root hair mutant phenotypes (Figure 5; see also Supplemental File 2). Interestingly, however, when *Ljcsld1-1* and *Ljcsld1-2* were crossed with either each other or with *Ljcsld1-5, Ljcsld1-7, Ljcsld1-8, Ljcsld1-9*, and *Ljcsld1-10*, a wild-type-like root hair phenotype was restored (Figure 5; see also Supplemental File 2).

### Expression and functional characterization of LjCSLD1

**Figure 6:**
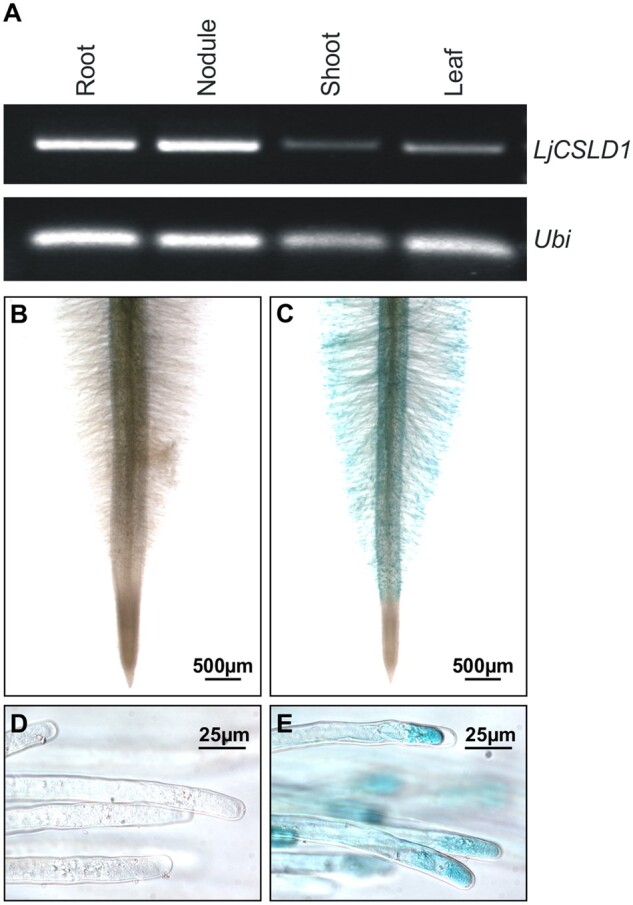
Expression of *LjCSLD1*. A, *LjCSLD1* mRNA is present in various *L. japonicus* tissues as assayed by RT-PCR. *Ubiquitin* mRNA (*Ubi*) was amplified and used as the RNA loading control. B–E, The *GUS*-reporter gene activity driven by the *LjCSLD1* promoter localizes to root hair tips. Wild-type (Gifu) plants were transformed with *A. rhizogenes* strain AR10 carrying the control binary vector containing the *LjCSLD1* promoter only (B and D), or the same vector with the *LjCSLD1* promoter transcriptionally fused to the *GUS* gene coding region (C and E). The resulting hairy roots were stained for the GUS activity and photographed.

The *LjCSLD1* mRNA was found to be present in all *L. japonicus* tissues tested, including uninoculated roots as well as nodules that formed upon inoculation with *M. loti* (Figure 6A). Histochemical analysis of the *LjCSLD1* gene expression in transgenic hairy roots carrying the corresponding promoter sequence fused to the coding region of the GUS reporter gene showed the promoter activity localized mostly in emerging to fully elongated root hairs of wild-type plants (Figure 6, B–E). These results were, therefore, consistent with the predicted role of the *LjCSLD1* gene in mediating root hair development in *L. japonicu*s.

To gain insight into the biochemical function of LjCSLD1, comparative chemical analyses of the root cell wall composition were performed. Although the use of only root hairs would have been preferable, this approach was not viable due to their overall scarcity in the mutant roots. Therefore, the entire roots of young wild-type and mutant seedlings were harvested and analyzed for monosaccharide content in cellulose and cell wall matrix polysaccharide fractions of the wall. In comparison with wild-type roots, cellulose was significantly diminished in the four variable root hair mutant lines (*Ljcsld1-2*, *Ljcsld1*-*4*, *Ljcsld1*-*6*, and *Ljcsld1*-*7*), while it was increased in *Ljcsld1-1*, the mutant line with the short root hair phenotype (Supplemental Table S4A). Analysis of neutral monosaccharides in the cell wall matrix (i.e. the noncellulosic fraction) indicated that compared with wild-type roots, the levels of mannose, as well as galactose, were lower in all mutants tested. While the content of other neutral sugars remained unchanged in *Ljcsld1-1* in comparison with wild-type samples, mutants of the variable root hair phenotype also had altered levels of fucose, arabinose, and xylose (Supplemental Table S4A). Consistent with these results, quantitative analysis of the hemicellulose fraction derived from independent root samples also revealed alterations in wall polymers, showing a significantly decreased level of mannose, relative to the wild-type control, in all *Ljcsld1* mutants tested (Supplemental Table S4B). No significant changes were detected in the corresponding pectic fraction (Supplemental Table S4B).

### Cell wall thickness is altered in *Ljcsld1-1* and *Ljcsld1-6* mutants

**Figure 7:**
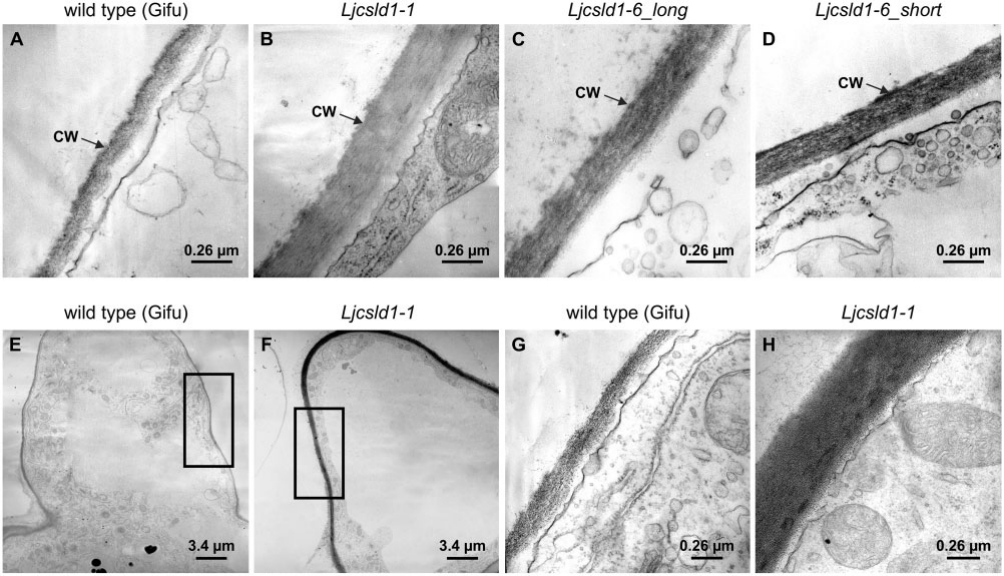
*Ljcsld1-1* and *Ljcsld1-6* alter root hair cell wall thickness and structure. A–D, Transmission electron microscopy (TEM) images of cell walls (CW) of root hairs of the mature root zone in *L. japonicus* wild-type (Gifu) and *Ljcsld1-1* and *Ljsld1-6* mutants. Note that *Ljcsld1-6* displays the variable root hair phenotype; therefore, longitudinal sections for two representative types of root hairs, short and elongated (long), that are formed by *Ljcsld1-6* were analyzed. E–H, In younger areas of the root, wild-type and mutant root hairs are similar in length yet the cell wall of *Ljcsld1-1* root hairs is almost three times thicker in comparison with the wild-type equivalent. The cell wall thickness was evaluated in the regions marked by rectangles in (E and F). Representative TEM images of longitudinal sections and the corresponding close-ups of root hair cell walls in *L. japonicus* Gifu (E and G) and *Ljcsld1-1* (F and H) are shown.

We further assessed the effects of the *Ljcsld1* mutations on root hairs by performing anatomical observations. Comparative analysis of root hair cell wall thickness between *Ljcsld1-1* (short root hairs), *Ljcsld1-6* (variable root hairs), and wild-type in the mature root zone, where wild-type root hairs have reached their final length, revealed that the mutants had significantly thicker cell walls than wild type (Figure 7, A–D). Observations performed on at least five independent root hairs belonging to at least five different individuals consistently demonstrated that *Ljcsld1-1* showed the largest increase, with an ∼0.37-µm cell wall versus the ∼0.15-µm cell wall of wild-type (Figure 7; Supplemental Figure S4). There was no difference in cell wall thickness between the short and longer root hairs of *Ljcsld1-6*, but they were both slightly yet significantly thicker than wild-type (Supplemental Figure S4). In order to mitigate, at least to some extent, possible effects caused by defects in root hair elongation, additional measurements were performed in a younger zone of wild-type and *Ljcsld1-1* roots, where root hairs of the former were actively elongating. In spite of a similar length, the cell wall of *Ljcsld1-1* root hairs was almost three times thicker than wild-type (Figure 7, E–H; Supplemental Figure S5).

### *Ljcsld1-1* has a semi-dominant effect on the root hair phenotype and affects root nodule development

**Figure 8:**
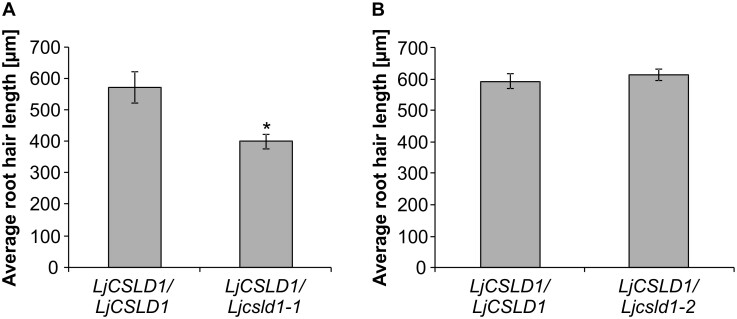
The *Ljcsld1-1* allele exerts a semi-dominant effect on root hair growth. F2 progeny derived from crosses between *Ljcsld1-1/Ljcsld1-1* and wild-type (*LjCSLD1/LjCSLD1*) (A) and *Ljcsld1-2/Ljcsld1-2* and wild-type (B) were genotyped and the root hair length was measured for homozygous wild-type (*n* = 12 and 22 for A and B, respectively) and heterozygous individuals (*LjCSLD1/Ljcsld1-1* and *LjCSLD1/Ljcsld1-2*; *n* = 41 and 51, respectively). The values represent averages ±95% CI. *P < 0.05 denotes statistically significant difference from the wild-type, homozygous genotype, as determined using a Student’s *t* test.

Since the *Ljcsld1* alleles developed thicker root hair cell walls, we evaluated the phenotypes of heterozygous plants in greater detail to see whether there were differences in root hair growth. F2 progeny derived from crosses between wild-type and either *Ljcsld1-1* or *Ljcsld1-2* were generated, and then the collective root hair length in the mature root zone (∼ 1 cm above the root tip) was measured, and each plant showing a wild-type-like phenotype was genotyped. There was no difference in root hair length between wild-type and heterozygous *LjCSLD1*/*Ljcsld1-2* plants. In contrast, a significant decrease in root hair length was observed in heterozygous plants of the *LjCSLD1*/*Ljcsld1-1* genotype, indicating that *Ljcsld1-1* has a semi-dominant effect (Figure 8).

**Figure 9:**
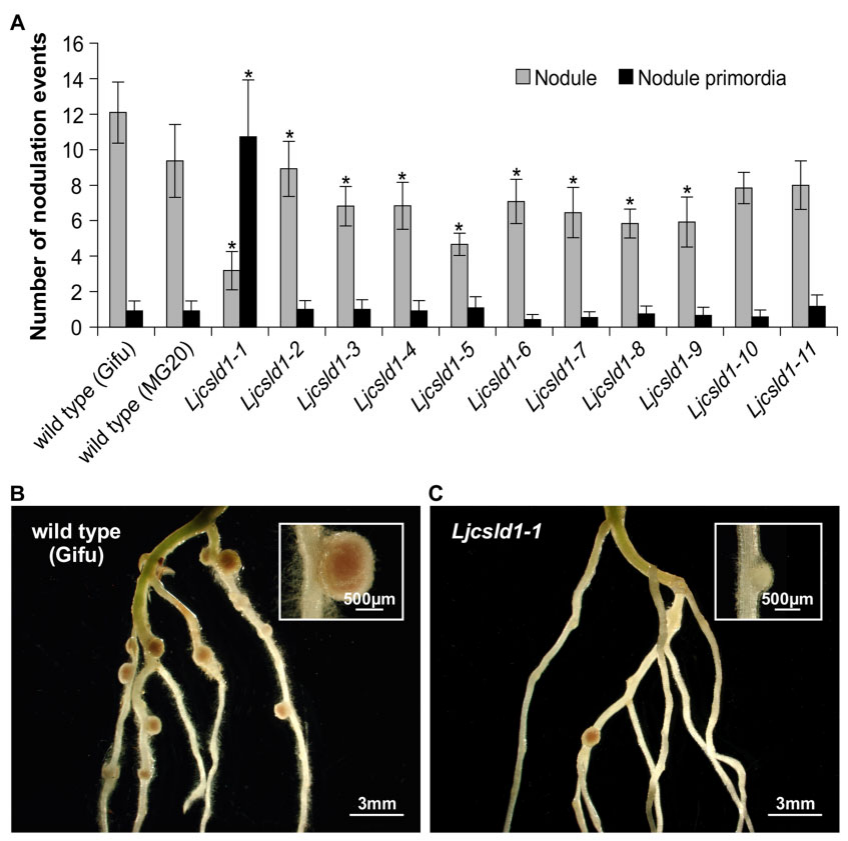
Symbiotic root phenotypes of wild-type *L. japonicus* Gifu and MG20 and the corresponding mutant plants carrying different *Ljcsld1* alleles; *Ljcsld1-1*, *Ljcsld1*-*2*, *Ljcsld1*-*4*, *Ljcsld1*-*6*, and *Ljcsld1*-*7* alleles are in the Gifu background, while the remaining alleles are in the MG20 ecotype. A, Numbers of nodules and nodule primordia 21 d after inoculation with *Mesorhizobium loti* strain NZP2235; an average number ± 95% CI is given for each genotype for *n* = 10–12. ^*^P < 0.05 denotes statistically significant difference from the wild-type, homozygous genotype, as determined using a Student’s *t* test. B and C, Representative images of roots and root nodules for (B) wild-type (Gifu) and (C) the *Ljcsld1-1* mutant. Note that all Gifu nodules are pink, while the majority of the mutant nodules are underdeveloped and white in appearance (see insets).

The observed differences, including cell wall thickness, between the short and variable root hair phenotypes, prompted the question of whether this corresponds with the severity of symbiotic phenotypes. When analyzed 21 d after inoculation with *M. loti*, the *Ljcsld1-1* line developed mostly uncolonized nodule bumps, while only a few nodules colonized by rhizobia were formed, confirming our previous data (Karas et al., 2005). In contrast, all mutant lines with the variable root hair phenotype formed large, colonized nodules, even though their nodule numbers were significantly decreased in most of the lines compared with wild type (Figure 9).

## Discussion

In this study, we identified a series of genetic lesions in the *L. japonicus CSLD1* gene that cause root hair developmental defects. All *Ljcsld1* mutations had a strong negative impact, resulting in root hairs that were much shorter than wild-type. However, except for *Ljcsld1-1*, all other mutant alleles appeared leaky, resulting in more variable root hair lengths. The semi-dominant character of *Ljcsld1-1* may account for this difference. Such an interpretation could be supported by the fact that the observed increase in the cell wall thickness, a shared phenotypic feature of *Ljcsld1-1* and *Ljcsld1-6*, was significantly more pronounced in *Ljcsld1-1*. However, the short root hairs of *Ljcsld1-1* and *Ljcsld1-6* differed in cell wall thickness, while both short and long root hairs of *Ljcsl1-6* did not differ significantly from each other in this respect (Supplemental Figure S4). Taken together, these observations suggest that increased cell wall thickness is unlikely to underlie defects in root hair elongation and most likely reflects a secondary effect. What this entails will need to be further investigated.

Previous evidence indicates that CSLDs homodimerize or multimerize like their CESA counterparts (Taylor et al., 2003; Yin et al., 2011; Li et al., 2018), and intragenic complementation has been reported for both CSLD and CESA proteins (Pysh, 2015; Li et al., 2018). This suggests that mutant proteins retain partial or regain full functionality as a result of dimerization/multimerization, or through participation in even larger hetero-complexes with other CSLD/CESA isoforms. The intragenic complementation and allele-specific phenotypes observed in our study are consistent with these earlier observations and also suggest modular functionality within the LjCSLD1 protein. The N-terminal domain of LjCSLD1 could function independently of the C-terminal portion of the protein, and within the N-terminus, the Cys-rich region could work independently of the PPTP, which is mutated in *Ljcsld1-1*.

The *Ljcsld1-1* mutation resulted in substitution of the third proline residue within the PPTP domain, which could impose a conformational change. This might lead to destabilization of the Ljcsld1-1-containing complex, therefore accounting for the semi-dominant nature of *Ljcsld1-1* and the strong, short root hair phenotype of the homozygous plants. Such an interpretation would be consistent with the observation that destabilization of even one CESA isoform is sufficient to cause degradation of the entire complex (Polko and Kieber, 2019). However, we cannot rule out the possibility that the *Ljcsld1-1* mutation has an opposite, stabilizing effect, resulting in overproduction of cell wall components with a detrimental effect on root hair growth. Distinguishing between these two scenarios will require detailed biochemical studies.

The *Ljcsld1-2* mutation, on the other hand, was in one of the seven of eight cysteine residues conserved with the two zinc finger domains present in AtCESA1 (Supplemental Figure S2B). For CESA, oxidation of the cysteine residues that form two zinc fingers within the N-terminus was shown to promote protein dimerization (Kurek et al., 2002). More recent evidence indicates that the N-terminal domains in CESA proteins interact to form cytoplasmic stalk structures involved in CESA–CESA and CESA-complex–microtuble interactions (Purushotham et al., 2020). It is possible, assuming cytoplasmic stalks also exist in CSLD complexes, that *Ljcsld1-2* could weaken CSLD1 interactions with each other and/or with microtubule-associated proteins (Li et al., 2018). Finally, the Ljcsld1-5, Ljcsld1-7, Ljcsld1-8, Ljcsld1-9, and Ljcsld1-10 and Ljcsld1-3, Ljcsld1-4, Ljcsld1-6, and Ljcsld1-11 proteins are likely to have either reduced or no activity, respectively. This was inferred based on the predicted impact of the mutations on the primary protein sequence and the phenotypic outcomes of the intragenic complementation experiments.

Intragenic complementation was observed for specific alleles. *Ljcsld1-1* and *Ljcsld1-2*, carrying N-terminal mutations, complemented each other and five other alleles (*Ljcsld1-5, Ljcsld1-7, Ljcsld1-8, Ljcsld1-9*, and *Ljcsld1-10*) that conferred defects in the C-terminal catalytic or transmembrane domains*.* In contrast, *Ljcsld1-3, Ljcsld1-4, Ljcsld1-6*, and *Ljcsld1-11* failed to complement *Ljcsld1-1* and *Ljcsld1-2*, possibly reflecting the severity of the mutations. For example, *Ljcsld1-6* and *Ljcsld1-11* both resulted in premature stop codons and might, therefore, be null mutations. This also suggested that other LjCSLD isoforms may also be involved with, but only marginally substitute for, LjCSLD1 during root hair formation, as reflected by the variable root hair phenotypes of the mutant lines. However, the presence of Ljcsld1-1 could interfere with this limited rescue effect due to its semi-dominant character. Interaction and activity studies are needed to test these various predictions.

To determine if our findings could be applied to other species, we performed a cross-species complementation experiment, which showed that AtCSLD2 and AtCSLD3 (KOJAK), required for root hair development in Arabidopsis (Bernal et al., 2008), are functionally equivalent to LjCSLD1. In *Populus trichocarpa*, PtrCslD5 is considered a functional orthologue of AtCSLD3 and, along with the highly homologous PtrCslD6, is involved in root hair development (Peng et al., 2019). Similarly, rice (*Oryza sativa*) OsCSLD1 is required for root hair morphogenesis (Kim et al., 2007). AtCSLD2 and AtCSLD3 have 90% and 92% sequence similarity, respectively, with LjCSLD1. Future research could focus on cross-species complementation using the other four AtCSLDs, which share 77%–84% sequence similarity with LjCSLD1. It would be particularly interesting to see whether AtCSLD1 and AtCSLD6, which lack the zinc finger regions, could complement LjCSLD1. Investigating whether introduction of the *Ljcsld1-1-*like proline mutation into other LjCSLDs or AtCSLDs would result in semi-dominant phenotypes should also be worthwhile.

We also analyzed cell wall composition using the entire roots of young wild-type and mutant seedlings. Glucose content in the cellulose fraction was significantly diminished in the four variable root hair mutant lines, which supports recent work that showed that Arabidopsis CSLD3 is a β-1,4-glucan synthase (Yang et al., 2020). Surprisingly, glucose content was increased in *Ljcsld1-1* mutant roots, and we currently do not have a good explanation for this effect. The *Ljcsld1-1* allele is semi-dominant and might be neomorphic or have some other properties that are less easily explained when compared with loss- or partial loss-of-function mutations. Regardless, since the assays were done using whole roots, these results support the observation that CSLD function is required for primary cell wall polysaccharide biosynthesis in addition to root hair formation (Li et al., 2009). The level of mannose was decreased in the hemicellulose fraction of root cell walls across all mutant genotypes tested, which is intriguing given the substantial differences in symbiotic and nonsymbiotic phenotypes between short and variable root hair mutants. It was previously suggested that mannose is enriched in soybean root hairs (Muszyński et al., 2015). Therefore, the simplest explanation is that roots with shorter or fewer root hairs would be predicted to have less of this component, and observed changes in levels of several other neutral sugars could also reflect root cell-specific differences in wall composition. But LjCSLD1 might also partake in complex interactions with different glycosyltransferases to mediate biosynthesis of various noncellulosic polysaccharides (Yin et al., 2011).

The difference between the semi-dominant and recessive alleles also corresponds with the nodulation phenotypes, with *Ljcsld1-1* having the most severe phenotype. During rhizobial infection of root hairs, the plant remodels the cell wall at the point of infection by expressing a nodulation specific pectate-lyase, which is required for the formation of the infection thread (Xie et al., 2012). It is possible that the thicker cell wall of *Ljcsld1-1* presents a stronger barrier to infecting rhizobia. Alternatively, LjCSLD1 may be required for the formation of the infection thread cell wall. Another possibility is that short root hairs are much less susceptible to infection because they cannot continue to grow and, therefore, to curl, which is required to capture bacteria and form a microcolony, from which the infection thread would be initiated (Fournier et al., 2015).

## Conclusion

We show here that the *LjCSLD1* gene, a likely ortholog of Arabidopsis *AtCSLD2* and *AtCSLD3*, mediates root hair development in *L. japonicus*, a legume plant. Results of intragenic complementation experiments suggest that LjCSLD1 performs this function by acting in a modular fashion as either dimers or multimers. The allelic series described here provides a unique resource for future detailed characterization of CSLD1 domains during cell wall formation.

## Materials and methods

### Plant material and growth conditions

*Lotus japonicus* root hair mutant lines S88-5A (*Ljsrh1)*, B12-IB (*Ljvrh1-1*), S49-AA (*Ljvrh1-2*), B69-A (*Ljvrh1-3*), and S36-1 were identified from a screen for genetic suppressors of the *L. japonicus* Gifu *har1-1* hypernodulation phenotype, as described (Karas et al., 2005; Murray et al., 2006b). Additionally, *L. japonicus* root hair mutants 210-226A, 212-010, 212-229, 01-0071, 212-164, and 212-447 were screened from ethyl methanesulfonate-treated M_2_ seeds of *L. japonicus* Miyakojima, MG20, at RIKEN Plant Science Center, and shared through the NBRP Legume Base resource.

Unless otherwise stated, all plants were germinated and grown as described in (Karas et al., 2005). For cell wall analysis, seeds (150 per genotype per replicate) were germinated and grown for 7 d on cellulose acetate film (Sterlitech Corporation, USA) moistened with water.

### Evaluation of root hair and symbiotic phenotypes

Symbiotic phenotypes were evaluated as described in (Karas et al., 2005). Root hairs of the F2 progeny derived from the crosses between wild-type and either *Ljcsld1-1* or *Ljcsld1-2* were measured for length by photographing a 2-mm section of each root at the root hair mature zone (∼1 cm above the root tip). To make a measurement, two vertical axes were drawn, one at the base of the root hairs and one at the edge of the root hair tips such that the second axis included 95% or more of photographed root hairs. Each measurement is the average distance between the axes. Measurements of root hairs were taken from both sides of each root and averaged.

### Identification of full-length mRNA and coding regions

The *LjCSLD1* cDNA was amplified by Reverse Transcription Polymerase Chain Reaction (RT-PCR) of the total RNA derived from *L. japonicus* roots using the coding region-specific primers (CSLD1_cDNAF and CSLD1_cDNAR; see List of all primers in Supplemental Table S5). Rapid Amplification of cDNA Ends (RACE) was subsequently carried out by using the First Choice RLM-RACE kit from Ambion (USA), according to the manufacturer’s instructions. The full-length *LjCSLD1* cDNA was reconstituted based on the obtained sequences. An additional set of two *LjCSLD1* mRNA-specific primers, positioned at the extreme 5′- and 3′-ends of the predicted full copy cDNA, were used (5′-outer race, 5′-inner race, 3′-outer race, 3′-inner race). Using total RNA from *L. japonicus* roots, RT-PCR was again performed and the resulting product was entirely sequenced.

### Expression analysis

Total RNA was isolated from *L. japonicus* tissues, and cDNA was synthesized as described previously (Murray et al., 2007). To evaluate steady-state levels of the *LjCSLD1* mRNA in different *L. japonicus* tissues, the following PCR conditions were used: 5 min at 94°C, 30 cycles of 94°C for 30 s, 60°C for 1 min, and 72°C for 30 s, followed by 7 min at 72°C (primers: LjCSLD1expF, LjCSLD1expR); the *Ubiquitin* cDNA was amplified (primers: Ubi-F, Ubi-R) using similar PCR conditions.

### Transgenic hairy roots

The 8567-bp genomic fragment encompassing the *LjCSLD1* gene was amplified with primers LjCSLD1compF and LjCSLD1compR from BAC clone LjT30G11 (National Bio-Resource project website: https://www.legumebase.brc.miyazaki-u.ac.jp/index.jsp). The resulting DNA was cloned into the pDONR221 vector (Invitrogen, USA) and subsequently moved to the pEarley303 destination vector. In addition, 6245-, 4804-, 4968-, and 4675-bp genomic fragments encompassing LjCSLD1, AtCSLD2, AtCSLD3, and AtCSLA9, respectively, were cloned into the pDONR221 and subsequently moved into the pEarley101 vector containing the *CaMV* 35S promoter (for primers, see Supplemental Table S5). For histochemical analysis (promoter expression studies), a 2605-bp fragment of the *LjCSLD1* promoter was amplified using LjCSLD1prmt_F and LjCSLD1prmt_R, and cloned into the pBI101 binary vector between XbaI and SmaI sites. The resulting constructs were transferred into *A. rhizogenes* AR10 strain and used to transform *L. japonicus* plants using the established protocol (Díaz et al., 2005). At least 20 independent plants were transformed for each construct analyzed. The resulting hairy roots were visually evaluated for the presence of root hairs. For the histochemical analysis of the reporter ß-glucuronidase (*gusA*) gene, transgenic hairy roots were stained as described previously (Held et al., 2014).

### Cell wall analysis

At 7 d after germination, roots were harvested from at least three biological replicates, cleared twice in a mixture of chloroform–methanol (1:1), and twice in acetone. Roots were subsequently air-dried, then weighed, and analyzed in triplicate. Cell wall composition was assessed by two independent methods (A and B). (A) Roots were treated with 1M sulfuric acid (H_2_SO_4_) (105°C, 1 h) to hydrolyze noncellulosic polymers. Hydrolysates were centrifuged to separate insoluble material, and after adding myo-inositol to each sample as an internal standard, the resulting soluble monosaccharides were derivatized to form alditol acetates (Blakeney et al., 1983) and analyzed by gas chromatography on an HP5880 gas chromatograph (Agilent, Santa Clara, CA) equipped with an SP-2330 column (0.25 mm × 30 m, Sigma-Aldrich Canada, Oakville, ON) and a flame ionization detector. The insoluble residue was incubated in a mixture of acetic acid: nitric acid: water (8:2:1, 105°C, 1 h), then washed with water and acetone and air-dried (Updegraff, 1969). The pellet was suspended in 67% (w/v) H_2_SO_4_, solubilized by shaking (23°C, 1 h), diluted to a final concentration of 1M H_2_SO_4_, and assayed for cellulose content by the anthrone method, as described by Updegraff (1969). (B) Cell walls from additional dried root samples were fractionated according to Fry (1988). Briefly, root tissue was extracted with 70% (v/v) ethanol (70°C, 1 h), and the alcohol insoluble residue was sequentially treated with dimethyl sulfoxide (DMSO): water (9:1, 23°C, 16 h) to remove starch, 0.5% (w/v) ammonium oxalate (105°C, 1 h followed by 4°C, 16 h) to extract pectins, and 4M potassium hydroxide (KOH) containing 0.1% (w/v) sodium borohydride (NaBH_4_)(23°C, 2 × 1 h) to isolate hemicelluloses.

Uronic acids (pectin) in the ammonium oxalate soluble supernatant were assayed based on the previously described method (Blumenkrantz and Asboe-Hansen, 1973). To one volume of supernatant, five volumes of 0.5% (w/v) borax solution in concentrated H_2_SO_4_ was added, mixed well, incubated (105°C, 5 min), then cooled in a water bath at 23°C. Absorbance at 520 nm (absorbance A) was measured. Subsequently, 1/10 volume, based on the original supernatant volume, of a 0.15% (w/v) solution of 3-phenylphenol in 1N sodium hydroxide (NaOH) was added, mixed well, and incubated (23°C, 5 min) before re-reading the absorbance at 520 nm (absorbance B). Uronic acid content was determined by subtracting absorbance A from absorbance B and comparing to a standard curve prepared using galacturonic acid.

Hemicelluloses in the KOH supernatant were assayed by neutralizing the extract with acetic acid, dialyzing against water to remove salts, and hydrolyzing to component monosaccharides in 1M H_2_SO_4_ (105°C, 1 h). Monosaccharides were converted to alditol acetates and quantified by the method of (Blakeney et al., 1983) as described in method **A**, above.

### Evaluation of root hair cell wall thickness

Roots segments were excised, fixed in glutaraldehyde and osmium tetroxide, and infiltrated in Epon/Araldite resin. In order to be able to obtain complete (from the tip to the insertion point) longitudinal sections of root hairs, we used a flat embedding procedure.

A glass microscope slide was covered with a thick layer of Teflon. Root segments were subsequently placed on the slide with a small drop of resin and covered with a Teflon coverslip. The slides were then incubated (60°C, 24 h) to allow resin polymerization. After removal of the Teflon coverslips, the slides were examined under a light microscope and the best points were selected and cut in order to obtain semi-thin and thin sections (only longitudinal sections were considered).

To compare cell wall thickness between wild-type, short (*Ljcsld1-1)*, and variable *(Ljcsld1-6)* mutants, we selected root hairs from the zone where they had reached their final length. Avoiding the apex and the basal regions, we obtained separate transmission electron microscopy images, at ×39,000 magnification, from 6 wild-type, 5 short, 7 variable “short type”, and 10 variable “long type” root hairs. Measurements taken at four different points along this imaged zone were averaged to obtain the cell wall thickness for a particular root hair. Additional measurements were obtained in the youngest portion of the roots, where hair length is similar for wild-type and the short root hair mutant, to exclude any possible effect of the degree of elongation on cell wall thickness.

### Phylogenetic analysis

The CSLD protein sequences of *L. japonicus* and Arabidopsis were aligned with ClustalW, and the corresponding phylogenetic tree was generated using MEGAX software and the neighbour-joining method with bootstrap replicates of 1,000.

### Accession numbers

Sequence data from this article can be found in the GenBank/EMBL data libraries under accession numbers: LjCSLD1 (LotjaGi3g1v0542600.1), LjCSLD2 (LotjaGi2g1v0450400.1), LjCSLD3 (LotjaGi6g1v0242300.1), LjCSLD4(LotjaGi2g1v0025800.1), LjCSLD5 (LotjaGi1g1v0761700.1), LjCSLD6 (LotjaGi2g1v0337200.1), AtCSLD1 (At2g33100), AtCSLD2 (At5g16910), AtCSLD3 (At3g03050), AtCSLD4 (At4g38190), AtCSLD5 (At1g02730), AtCSLD6 (At1g32180).

## Supplemental data

**Supplemental Figure S1.** Map-based cloning of the *LjVRH1*/*LjSRH1* locus.

**Supplemental Figure S2.** The LjCSLD1 protein contains several conserved domains.

**Supplemental Figure S3.** Alignment of predicted CSLD protein sequences of *L. japonicus* and Arabidopsis.

**Supplemental Figure S4.** *Ljcsld1-1* and *Ljcsld1-6* significantly increase cell wall thickness of mature root hairs.

**Supplemental Figure S5.** *Ljcsld1-1* significantly increases the root hair cell wall thickness.

**Supplemental Table S1.** List of *L. japonicus* mutant lines and corresponding *Ljcsld1* alleles.

**Supplemental Table S2.** Identity and similarity values for comparison of full length *L. japonicus* and Arabidopsis CSLD proteins.

**Supplemental Table S3.** Additional information about LjCSLD1 genomic, mRNA and protein sequences.

**Supplemental Table S4.** Analyses of cell wall composition in *L. japonicus* wild type and *Ljcsld1* mutant roots.

**Supplemental Table S5.** List of primers used in this study.

**Supplemental File S1.** Sequences:

*L. japonicus* GIFU Cellulose Synthase-like D1: genomic sequence intron-exon structure.

*L. japonicus* GIFU *Cellulose Synthase-like D1*: mRNA.

**Supplemental File S2.** Intragenic complementation for all allelic crosses

## Supplementary Material

kiab204_Supplementary_Data

## Notes

B.J.K and K.S. conceived the original screening and research plans; B.J.K and K.S. supervised the experiments; B.J.K, L.R., and L.A performed most of the experiments; B.J.K, L.R., M.No., L.A., A.S., S.S., D.B., J.D.M, P.B., and K.S. designed the experiments and analyzed the data; B.J.K and K.S. conceived the project and wrote the article with contributions from all of the authors; B.J.K. and K.S. supervised and complemented the writing. S.I, M.Na., and T.S. provided seeds for the following mutant lines: 210-226A, 212-010, 212-229, 01-0071, 212-164, and 212-447; S.S. provided *L. japonicus* genome sequence data and identified *L. japonicus* CSLD gene family; P.B. and M.Na performed the transmission electron microscopy experiment.

The author responsible for distribution of materials integral to the findings presented in this article in accordance with the policy described in the Instructions for Authors (https://academic.oup.com/plphys/pages/general-instructions) is: Bogumil J. Karas (bkaras@uwo.ca).
