# Supplementary material for: Intragenic complementation at the Lotus japonicus CELLULOSE SYNTHASE-LIKE D1 locus rescues root hair defects
Source: Plant Physiol. 2021 May 6;186(4):2037–50. doi: 10.1093/plphys/kiab204 (PMC8331140; doi:10.1093/plphys/kiab204)
Supplement: kiab204_Supplementary_Data [file kiab204_supplementary_data.zip › pp.00250.2021-s01.pdf]

SEQUENCES

***L. japonicus* GIFU Cellulose Synthase-like D1: genomic sequence intron-exon structure**

Promoter (bold)

5’ and 3’ UTRs: underline

Predicted start and stop codons: grey highlight

Exons: UPPERCASE

Intron sequences: lowercase

TTTTTGTTTTGGTCAACAGCAGTATAATCAGCTTTATAGCTAATTTCATGGGCA
TTTCTTTTGTTAGGCCTTATTTGCGCCAAAAAAGTTTTTCAAACATAGAATAATT
TTTCAATTTTCATCTATGTTCTTAGTACAAGTTAATAGGCACATTGAATATGTTG
GGTCATCACAATCTCATGATCTCATTTATTATTGTCATGTCACCATCCCTCCATC
TCAACTTGTTGGTCCACAAACAGCTGAATTGAAATGGGTGAGATTTTGATTGGA
AAAAATAGCAGCACTGAGACCATCAAAACCGGCGAGATTTCAGACCATTTTTG
GCATAACACATTGGAATGAGAACTAAAAAGAATAAACAACACCATCCAAGTCAA
GAGTGGAGCCAGAGGACCATGGGCAAAAGTAATCAGCAGGAAATAAACGACAG
TAGAGACAGAATATTCCCATTCTTGATGAGTACTATAGCTTTCCATTAAGCGGA
GCACGTTTTTTCTTTTTATGATGACAAAGCAAGGGAAAGGGAAACAAACAGTAC
TAATGCACCGATGTTTCTACATATAATACATGGTATAAATGTTAACTACTTTACA
CAAGGAATTTTGACAAACTTCCCTTGTGTTATTTGTGCATTTCTCGAATTTCCTT
TCCTTGAAGTTAGGGGTGGAAATAGGTCAGACGGCTCGTCAGGGGCCTATGGC
TTGGCTCGTTAGAGGCTCGGCTCAGCTCAGCTCGTTTATTAAAAAGGCCAGGC
TTAGGCTTTTTTAAAAGTTTGTTTAACTAAAAAGGGCAGGCCCAAACTATTAAA
AAAGCCTATTTGGCCTAACAGGCCGGCCTATTTATATATTATTTGATTTTTTAAA
ACTGATGTATAGTTTAGTATAAAAGAAAACCCCAAGTCCCTACCCTAACCTAAA
TTAGAAACGTTACAGACATATTCAATTATACAGATTTAGTTTTTGAAATGCAAT
GATACAGGTTTTTTTATTTGAAAAGAAAGTGCAATGATATATAAAAGGCTTATT
AGCCCGCCAGCCTATTCTTTTGATAAAATTGTCTGTTAATAGGCTTTTAAGCAG
GCTTGTAGACCAAGCCAGGCCTTAGAAAAAGCCAGGTCAAGCCACAAAATTTG
GTCTATTGATAGGCCACAGGCCAGACTTGGGCCACGAAAAGATAATGCAGGCC

AGGCCTAGGCCACGTAAAGCTTGGCTCGGCTCGGCCTATTTCCACCCCTACCC
CTACTTGAAGTTGAAGTAAACAAACCATGGTTTGTTCTATTCAAAGTTTCAAAC
AAAGCTTACCCCTTGATAAATTTGGAACGATAAGCAATGAATTGATAGTATGAA
ATGAAACTATATAATCAAATATCACATTTGACTTGACAGCATCAATAGCGTGTT
ACGTCTTATCTTAAGCAAATGTAAAGTATATGATAGATAACAATATAAATAAGA
AGATAAAACGCGTTTGTTTCCCATTACCATAAGACAACCATGTGATGCGAGTTG
CTCCTTTATTACAGCACATATGTCGTGTTCTGTTTTGAGGGTGTTTTGGGAATC
AAGAGATCCTACAATGTAACGAGGGAAAGACATTTTGAACTTTGAAGATCCAAA
ACAAAAGGAAAAAAAATAAAATATGATAAGTGTGGGTTCAGATTTTCATTCAAC
TCAACCTGAATTCCTCTTGAAGGCAACGTGTTAAAAACATGCTTCTCTCATTAA
ATTTAATGTGTCAAGTCAAATTTTATCTTCTCTGTTAGTATCTGCCTTAAGTGTG
AAATCACATGTACATTAACTCAAACACCAAAATAAACATACAATAAATTAAATA
CGGATGTATATGATAAAAATTGGGAAGACGGCAGACACATGTTGCCTGGTCAT
GACAGCAAGTAATGGCTTAACGTGTCAACGACATTGCTCCCAGTTTCCTTAAAC
TAAATAAAAATTCTTGTATTGATTTCGTCAAAATTCTTCATTAGGCATTCATAGA
GTTTAAATTTTCCAATTTTATTACTCTCTATAAATCATAATAAATAATAATAATA
TACTTATACTCTGTAAATGATTTAACAGCAGTGATGGTATGATAAATTCCTATA
TACTTAATTTTTTAATAAGATAAAGGCCTATTTTTATGGTTAAAAAAAGCTACTT
TGAAGTAAAAAAATCAGAATATTCATTTCAAAAAAATATCAGAATATTACTACA
CTACACAAAAAATAGTCTTAAAAAAATCTACTGCCATCAATTCAACCCCACTTC
TTTCTCAAATCCAATAACCTTCGGACAATTTTGTATTTCTCGTAAGAAGATTTAG
CGATATACCCACAAGGTAGTTTAGTTGGCAACGTAAGTTGAGTGCGGAAAGAA
CTTTATAATCCCTAACATTAAGAGAATGGATGAAAAGTCTTAATTACGACTAAA
TTTCAAATCCATAATAGTCAACATTTAAAGTGTGACGGACTACCAGAGTTTTTA
TTAAAAGAAAATTAAGAATGAATTATGAATATGATATGAATGCTCCAATCATGG
CCGGTAGTGCTTGAAGTTTTGCTCACGACTAACTACACTTCTCTCTCTCTCCGT
GTGCCAGTGTATGTTTTTAAGGTGACAATTGGAGACCACATCT<u>AACTTCTTACAC</u>
<u>TCTTTGAAGCTTTCAAATACTCTGTTTTCATGTCATGGCTTTCTTATAAGATCACAAC</u>
<u>TCAGAACACAGATCTCAAACTGAGTGGCATTTAGTTTTTCAAACAGCAG</u>gtactgttcctttct
ctgagctaatgcattcatggggtgcttttttccaataagaaaatctactttttggtttctagttaagctttttatgattgtgatgactttcttgcatatgt
agcgggttgattgattgccgagaatataggcgagacatgtgaaaatatgggaattatgaatgatttttatgacaatggacttctttctaggatttg

tagattttttcaaaggttaagcaatgaatggaactcaaagggatattttttattttctctactttgttcttcttggaaaccaagagtcatctatgaagg
attttgaactttttcatcagatatgatccttctgtgagcttattgagttaaacattttcatcttagaagagttttggatcttgaacttggggtttaagga
acaaaatgtccattagttgagtgtctgcatctgtgtactctagattcaggtgaatatgattgctttttaaaggttagttatatcagcttcttcttggtca
gaatcaattctaaaacggataagctactcacagaagcttctccctaaaattgattctgacttcataatcaattggagaagagtttcctaacatgta
gttagattaaggaacagtctgttgctttttccttggtatttcagaaaaattcttcacattttctgtaaattcttcagaagtttactgagatctaatgcttt
gccctttgaatctgtctgaaacgtttcgttacattattctttttaataatagatttgaaaagcttggacttgattgagacttgaataatgtgtctttattat
ttggtatagttctcttgaatatttctttcttttatcatggcactttgaagtattgattaacaattttaacattcaccttgattttggtttaactctattaactttt
cattttggaatcaattacaatgctaatgaagatctgatttgctagagttttgaatttcagGCTATTCTGATTAGTCTTGCTTT
GTCCTCAAATATGGCATCAAAACCATTCAAACAAAGCCGGTCATCTCTTTCAGCAGC
TTCTGATGCATCTGAAGCCCAGAAGCCTCCTTTACCTCCAACTGTAACATTTGCCCG
AAGAACTTCCTCGGGGCGCTACGTCAATTACTCCAGGGATGATCTTGACAGTGAGCT
GGGAAGTGAGCTAGGAAGTACTGATTTCATGAATTACACAGTGCATTTACCACCAAC
CCCTGATAACCAACCTATGGATTTAACAGTCTCACAGAAAGTTGAGGAGCAATATGT
ATCAAACTCGCTATTTACCGGAGGATTCAACAGCATGACTAGAGCCCATCTAATGGA
TAAGGTGACAGAATCTAAAGCAAACCATCCACAGATGGCTGGTGTAAAAGGGTCTT
CATGTGCAGTTCCTGGTTGTGATTGTAAAGTGATGAGCGATGAACGCGGTGAGGATA
TTCTTCCTTGTGAGTGTGATTTTAAGATATGTAGAGACTGCTATATAGATGCAGTGA
AAACAGGAGATGGAATGTGCCTAGGATGCAAAGAGCCATATAAGAACACAGAGCT
AGATGAAGTGGCTGTGGATAATGGAAGGTCATTTCCACTTCTTCCGCCAAATGGGGG
AGTGTCGAAAATGGAGAGGAGATTGTCCTTGATGAAGTCAACAAAATCAGCACTGA
TGAGAAGTCAAACTGGAGATTTTGATCACAATAGGTGGCTCTTTGAAACAAGGGGT
ACCTATGGCTATGGAAATGCAATTTGGCCAAAGGAAGGGAATTTTGGAAATGGAAA
AGAGGATGGTGATGTTGTTGACCCAACTGAGTTGATGAACAAACCCTGGAGGCCAC
TTACAAGGAAACTCAAGATACCTGCTGCTATTCTGAGTCCATATCGgtacaaaaccttttgtcttt
gatgctgtttacttttaagataaatttttttttggttaatgccataaatgttgtttttggtcataccaagttttctactaatcaaaccatgaacttacctttt
gttctgaatggccatttggcttcattatacctgaccctatcggaatgcaatatttcatgattggggaattttataatatttcaaggttgtgttaggcgt
gcaacattggatccacaaagtgggttaggcccctctctggaccttgtgcatagcgggagctttatagtaccgggtttgcccttttttgtgttaggc
atgcggcagccaacgtaaaattagtcgtatatttaatatggatcagccgacctcacttagtgggataagacttttgttgtgttgtcttttgcaatga
tgagatgattggttttgatagaaatgtgctctggactctatttaaatgaatagcatataaaggggcaaaaagcatgattgatggagtttttcattta
gattattttgatagaagaatggaaattggttttgatgtttggctattgtaccttagttttgaaggaacaatatggtttccatctgtgtattagatcctga
ttatgtttatgcagtccttaacactgccaaacttgcagtaatatattagcttgtttaactttataacattgcctatagttctataatgtattatattcatga

acataaagaataatgttccttaaacaaaaatctatgtcttctaacaattgtatttctttctgttgcatctggttttacagTCTCATCATTTT
GGTTCGCTTCGTTATCCTAGTCCTGTTCCTGGAATGGAGGATCAGGCACAAAAATAC
TGATGCAATCTGGCTATGGGGTATGTCTGTGGTTTGTGAGTTATGGTTTGCTTTTTCT
TGGCTTCTGGATCAACTTCCCAAGCTGTGCCCGATAAATCGCTCAACGGATCTTAAT
GTTCTGAAGGAAAAATTTGAAACACCAAGTCCTACCAATCCTACTGGAAAGTCTGAT
CTGCCAGGAATAGATATCTTTGTATCTACTGCAGATCCTGAGAAAGAACCACCTCTT
GTCACTGCAAACACTATCTTGTCTATTTTAGCTGCTGATTATCCAGTTGAGAAGCTTT
CTTGCTATGTTTCTGATGATGGAGGTGCACTTCTAACTTTTGAGGCAATGGCTGAAG
CTGCCAGTTTTGCTAATGTATGGGTTCCATTCTGTCGTAAACATGATATAGAGCCTA
GAAATCCTGAATCATATTTCAGCTTGAAGCGAGACCCTTACAAGAACAAAGTGAAA
CCAGATTTTGTGAAGGATCGTAGACGGGTGAAGCGCGAGTATGATGAGTTCAAGGT
CAGAATCAATGGTTTGCCTGACTCTATTCGCCGCCGGTCAGATGCCTTTCACGCAAG
AGAGGAAATCAAGGCCATGAAACTTCAGAGACAGAACAAGGAAGATGAACCTATA
GAAGCTGTAAAGATTCCAAAAGCAACATGGATGGCTGATGGAACTCATTGGCCAGG
GACTTGGTTGAACTCCTCAGCTGAGCATTCTAAGGGTGACCATGCTGGTATAATTCA
Ggtactgaggctgtgtttgaaagagtttatttgagtttacttatagtaaaaacacttatgcaagtgtttggataaacttatgaacataatttatgatct
gcctacaagctcttttgggattattttcataagtttctcataatagcttatgaataagcacttatacatacctaaaacagttttgagcttaagttcaata
aacttctcaaagaagcttatgaatatgtgattagtgcttattgtcataatcacttaattaagttgtttacctaaatatactcaagtgtatgttaggttta
gcattggagccagtccaaaaccatgatgttagcaaaaactcttgtccattagcttttgctcagaccatgttgttaggattcttgggattcccaatg
taaagtcaaactcactataagtgcatatttggttccacggtttgcacagaatccaaaacacatttaagctaagacttgtggcttctaacaagaag
tgattcaagagcatttcaccttggaaccaaacatgttataagtagtttttgtaggagtaatatccaagtgtgaagatgagagtaaataactcatatt
tgatgaatgatacagGTGATGTTGAAACCTCCTAGTGATGAACCTCTTCTTGGAAATGATGAT
GATACAAAGCTCATTGACCTGACTGATATTGATATCCGTCTTCCCCTTCTTGTCTACG
TTTCTAGAGAGAAACGTCCAGGCTATGATCACAACAAAAAAGCAGGTGCCATGAAT
GCCTTGGTCAGAGCCTCAGCCATCATGTCCAATGGTCCTTTTATACTCAACCTTGACT
GTGACCACTACATCTACAACTCGAAGGCAATGAGGGAAGGCATGTGCTTTATGATG
GACCGTGGTGGCGACCGCCTTTGCTACGTTCAGTTCCCACAGAGGTTTGAAGGGATT
GATCCCTCTGATAGATATGCTAATCACAACACTGTCTTCTTTGATGTAAACATGAGA
GCCCTTGATGGTCTTCAAGGGCCAGTGTATGTAGGAACTGGTTGTCTTTTCAGAAGG
GTTGCCCTTTATGGATTCGATCCTCCCCGAGCAAAAGAACACCATCCAGGTTTCTGC
AGTTGCTGTTTTGGAAGGCGCAAGAGAATTGCTAGCCACAACACTGAAGAGAACCG

AGCGCTGAGGATGGGTGATGATGACTCTGAGGATGAAGAAATGAACCTGTCAACGT
TCCCTAAGAAGTTTGGGAACTCAACTTTCCTCATTGAATCAATCCCAGTGGCAGAGT
TCCAAGGCAGGCCACTCGCTGATCACCCTGCTGTGAAAAATGGGCGCCCTCCCGGTG
CTCTCACCATACCCCGCGAGCTTCTTGATGCAGCAACCGTGGCAGAGGCCATCAGTG
TGATCTCATGTTGGTATGAGGACAAGACTGAGTGGGGGCAGCGCGTCGGATGGATC
TACGGATCAGTCACTGAGGATGTTGTCACCGGTTATAGAATGCACAACCGAGGATG
GAAATCAGTTTACTGTGTGACAAAGCGTGATGCTTTTCGCGGTACTGCTCCCATCAA
CCTCACTGATAGGCTGCATCAAGTTCTTAGGTGGGCTACAGGTTCAGTTGAGATATT
CTTCTCCAGAAACAATGCATTTCTTGCAAGCCCCAGAATGAAAATCCTTCAAAGAAT
AGCATACCTTAATGTTGGAATCTATCCATTCACTTCTTTTTTCCTCATTGTCTACTGCT
TCCTCCCTGCACTTTCCCTCTTCTCAGGCCAGTTCATTGTTCAAACACTCAGCGTCAC
TTTTCTCTCTTATCTATTAGGCATCACAGTGACACTGTGCATACTTGCTGTGCTTGAG
ATTAAATGGTCAGGGATTCAGCTTGAAGAATGGTGGAGGAATGAGCAGTTTTGGTT
GATTGGAGGGACCAGTGCTCATTTAGCTGCTGTGCTTCAAGGACTGCTCAAAGTGAT
AGCAGGCATTGAAATCTCCTTCACCTTGACCTCAAAATCTGGTGGTGATGATGTCGA
TGACGAGTTTGCTGATCTCTATGTTTTCAAGTGGACATCGCTCATGATACCACCCATC
ACAATCATGATGGTTAACTTGATAGCAATTGCAGTCGGAGTGAGCAGGACCATATA
CAGCACCATACCTCAGTGGAGCCGTTTGCTAGGTGGTGTTTTCTTCAGCTTTTGGGTG
CTGACTCATCTCTACCCTTTTGCTAAAGGTTTGATGGGAAGAAGAGGGAGGACACCT
ACCATTGTTTATGTGTGGTCAGGTCTCATAGCAATCACAATATCACTCCTTTGGGTTG
CAATCAATCCCCCTCAAGGTGCCAACGAGATAGGTGGTTCATTCCAGTTCCCATGAT
AGCTCATTCTTTTTGTCATATGACAATTTCTTTCTTTTGTTTTTTGTGTAAAATTCTTTT
TCCCTCTTAAGTCATCAACATGATGATAATAGTGAATGGACCTAAATATTACTCTGT
TTATTGAGCTTATGAATTTTGTTCAGATAGTGAATCCCTGGAATCAGGAACGTTTGA
TGGTGAATTGAAATAACTTTCTTATTCC

L. japonicus **GIFU** ***Cellulose Synthase-like D1*****: mRNA**

Underlined sequences: 5’ and 3’ UTRs

AACTTCTTACACTCTTTGAAGCTTTCAAATACTCTGTTTTCATGTCATGGCTTTCTTAT
AAGATCACAACTCAGAACACAGATCTCAAACTGAGTGGCATTTAGTTTTTCAAACAG

CAGGCTATTCTGATTAGTCTTGCTTTGTCCTCAAATATGGCATCAAAACCATTCAAAC
AAAGCCGGTCATCTCTTTCAGCAGCTTCTGATGCATCTGAAGCCCAGAAGCCTCCTTT
ACCTCCAACTGTAACATTTGCCCGAAGAACTTCCTCGGGGCGCTACGTCAATTACTCC
AGGGATGATCTTGACAGTGAGCTGGGAAGTGAGCTAGGAAGTACTGATTTCATGAAT
TACACAGTGCATTTACCACCAACCCCTGATAACCAACCTATGGATTTAACAGTCTCAC
AGAAAGTTGAGGAGCAATATGTATCAAACTCGCTATTTACCGGAGGATTCAACAGCA
TGACTAGAGCCCATCTAATGGATAAGGTGACAGAATCTAAAGCAAACCATCCACAGA
TGGCTGGTGTAAAAGGGTCTTCATGTGCAGTTCCTGGTTGTGATTGTAAAGTGATGAG
CGATGAACGCGGTGAGGATATTCTTCCTTGTGAGTGTGATTTTAAGATATGTAGAGAC
TGCTATATAGATGCAGTGAAAACAGGAGATGGAATGTGCCTAGGATGCAAAGAGCC
ATATAAGAACACAGAGCTAGATGAAGTGGCTGTGGATAATGGAAGGTCATTTCCACT
TCTTCCGCCAAATGGGGGAGTGTCGAAAATGGAGAGGAGATTGTCCTTGATGAAGTC
AACAAAATCAGCACTGATGAGAAGTCAAACTGGAGATTTTGATCACAATAGGTGGCT
CTTTGAAACAAGGGGTACCTATGGCTATGGAAATGCAATTTGGCCAAAGGAAGGGAA
TTTTGGAAATGGAAAAGAGGATGGTGATGTTGTTGACCCAACTGAGTTGATGAACAA
ACCCTGGAGGCCACTTACAAGGAAACTCAAGATACCTGCTGCTATTCTGAGTCCATAT
CGTCTCATCATTTTGGTTCGCTTCGTTATCCTAGTCCTGTTCCTGGAATGGAGGATCAG
GCACAAAAATACTGATGCAATCTGGCTATGGGGTATGTCTGTGGTTTGTGAGTTATGG
TTTGCTTTTTCTTGGCTTCTGGATCAACTTCCCAAGCTGTGCCCGATAAATCGCTCAAC
GGATCTTAATGTTCTGAAGGAAAAATTTGAAACACCAAGTCCTACCAATCCTACTGG
AAAGTCTGATCTGCCAGGAATAGATATCTTTGTATCTACTGCAGATCCTGAGAAAGA
ACCACCTCTTGTCACTGCAAACACTATCTTGTCTATTTTAGCTGCTGATTATCCAGTTG
AGAAGCTTTCTTGCTATGTTTCTGATGATGGAGGTGCACTTCTAACTTTTGAGGCAAT
GGCTGAAGCTGCCAGTTTTGCTAATGTATGGGTTCCATTCTGTCGTAAACATGATATA
GAGCCTAGAAATCCTGAATCATATTTCAGCTTGAAGCGAGACCCTTACAAGAACAAA
GTGAAACCAGATTTTGTGAAGGATCGTAGACGGGTGAAGCGCGAGTATGATGAGTTC
AAGGTCAGAATCAATGGTTTGCCTGACTCTATTCGCCGCCGGTCAGATGCCTTTCACG
CAAGAGAGGAAATCAAGGCCATGAAACTTCAGAGACAGAACAAGGAAGATGAACCT
ATAGAAGCTGTAAAGATTCCAAAAGCAACATGGATGGCTGATGGAACTCATTGGCCA
GGGACTTGGTTGAACTCCTCAGCTGAGCATTCTAAGGGTGACCATGCTGGTATAATTC
AGGTGATGTTGAAACCTCCTAGTGATGAACCTCTTCTTGGAAATGATGATGATACAA

AGCTCATTGACCTGACTGATATTGATATCCGTCTTCCCCTTCTTGTCTACGTTTCTAGA
GAGAAACGTCCAGGCTATGATCACAACAAAAAAGCAGGTGCCATGAATGCCTTGGTC
AGAGCCTCAGCCATCATGTCCAATGGTCCTTTTATACTCAACCTTGACTGTGACCACT
ACATCTACAACTCGAAGGCAATGAGGGAAGGCATGTGCTTTATGATGGACCGTGGTG
GCGACCGCCTTTGCTACGTTCAGTTCCCACAGAGGTTTGAAGGGATTGATCCCTCTGA
TAGATATGCTAATCACAACACTGTCTTCTTTGATGTAAACATGAGAGCCCTTGATGGT
CTTCAAGGGCCAGTGTATGTAGGAACTGGTTGTCTTTTCAGAAGGGTTGCCCTTTATG
GATTCGATCCTCCCCGAGCAAAAGAACACCATCCAGGTTTCTGCAGTTGCTGTTTTGG
AAGGCGCAAGAGAATTGCTAGCCACAACACTGAAGAGAACCGAGCGCTGAGGATGG
GTGATGATGACTCTGAGGATGAAGAAATGAACCTGTCAACGTTCCCTAAGAAGTTTG
GGAACTCAACTTTCCTCATTGAATCAATCCCAGTGGCAGAGTTCCAAGGCAGGCCAC
TCGCTGATCACCCTGCTGTGAAAAATGGGCGCCCTCCCGGTGCTCTCACCATACCCCG
CGAGCTTCTTGATGCAGCAACCGTGGCAGAGGCCATCAGTGTGATCTCATGTTGGTAT
GAGGACAAGACTGAGTGGGGGCAGCGCGTCGGATGGATCTACGGATCAGTCACTGA
GGATGTTGTCACCGGTTATAGAATGCACAACCGAGGATGGAAATCAGTTTACTGTGT
GACAAAGCGTGATGCTTTTCGCGGTACTGCTCCCATCAACCTCACTGATAGGCTGCAT
CAAGTTCTTAGGTGGGCTACAGGTTCAGTTGAGATATTCTTCTCCAGAAACAATGCAT
TTCTTGCAAGCCCCAGAATGAAAATCCTTCAAAGAATAGCATACCTTAATGTTGGAAT
CTATCCATTCACTTCTTTTTTCCTCATTGTCTACTGCTTCCTCCCTGCACTTTCCCTCTT
CTCAGGCCAGTTCATTGTTCAAACACTCAGCGTCACTTTTCTCTCTTATCTATTAGGCA
TCACAGTGACACTGTGCATACTTGCTGTGCTTGAGATTAAATGGTCAGGGATTCAGCT
TGAAGAATGGTGGAGGAATGAGCAGTTTTGGTTGATTGGAGGGACCAGTGCTCATTT
AGCTGCTGTGCTTCAAGGACTGCTCAAAGTGATAGCAGGCATTGAAATCTCCTTCACC
TTGACCTCAAAATCTGGTGGTGATGATGTCGATGACGAGTTTGCTGATCTCTATGTTT
TCAAGTGGACATCGCTCATGATACCACCCATCACAATCATGATGGTTAACTTGATAGC
AATTGCAGTCGGAGTGAGCAGGACCATATACAGCACCATACCTCAGTGGAGCCGTTT
GCTAGGTGGTGTTTTCTTCAGCTTTTGGGTGCTGACTCATCTCTACCCTTTTGCTAAAG
GTTTGATGGGAAGAAGAGGGAGGACACCTACCATTGTTTATGTGTGGTCAGGTCTCA
TAGCAATCACAATATCACTCCTTTGGGTTGCAATCAATCCCCCTCAAGGTGCCAACGA
GATAGGTGGTTCATTCCAGTTCCCATGATAGCTCATTCTTTTTGTCATATGACAATTTC
TTTCTTTTGTTTTTTGTGTAAAATTCTTTTTCCCTCTTAAGTCATCAACATGATGATAAT

AGTGAATGGACCTAAATATTACTCTGTTTATTGAGCTTATGAATTTTGTTCAGATAGT
GAATCCCTGGAATCAGGAACGTTTGATGGTGAATTGAAATAACTTTCTTATTCC
