# Supplementary material for: Intragenic complementation at the Lotus japonicus CELLULOSE SYNTHASE-LIKE D1 locus rescues root hair defects
Source: Plant Physiol. 2021 May 6;186(4):2037–50. doi: 10.1093/plphys/kiab204 (PMC8331140; doi:10.1093/plphys/kiab204)

Supplemental file 2 – Intragenic complementation for all allelic crosses.
Note: This figure re-uses the images representing each parental genotype for illustrative purposes.

Parent 1	Parent 2	F1

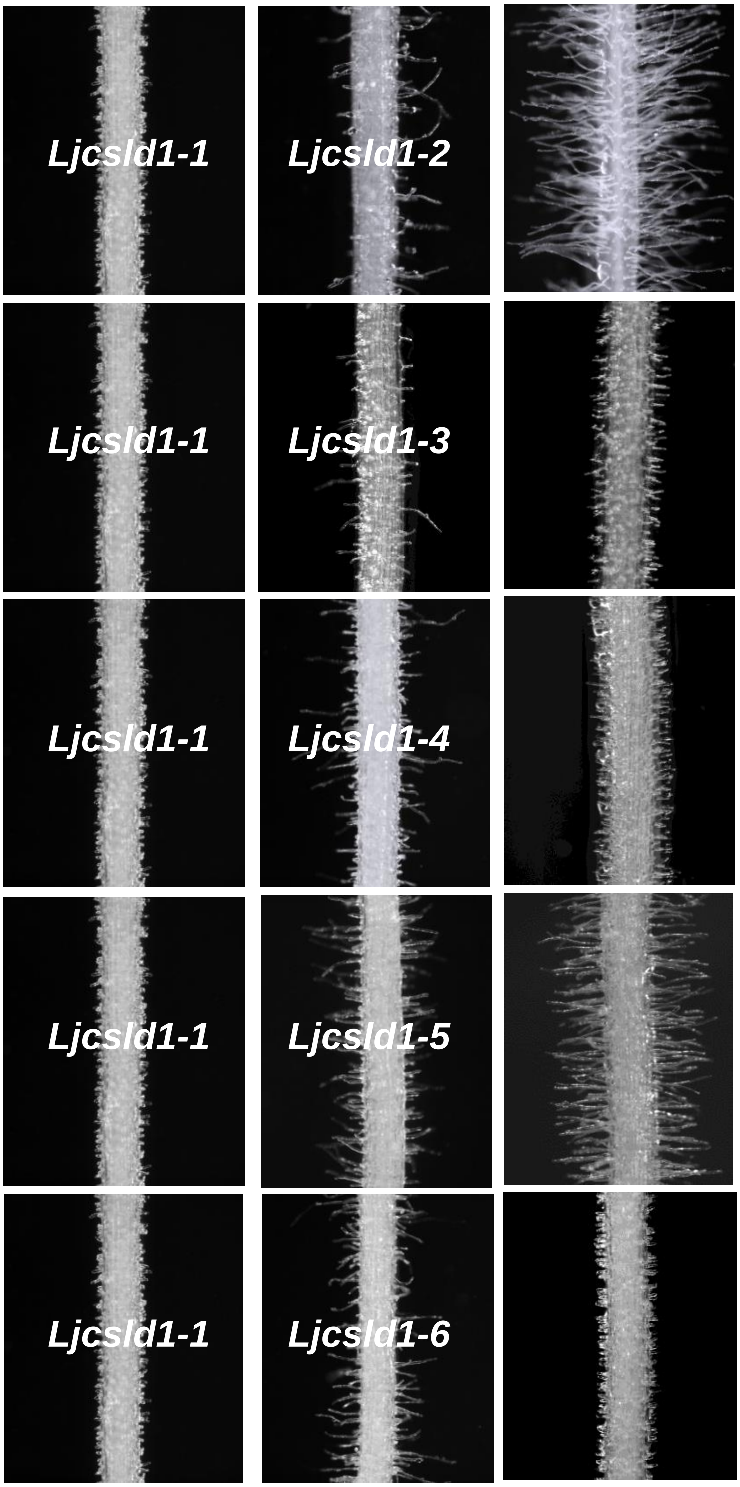

Parent 1	Parent 2	F1

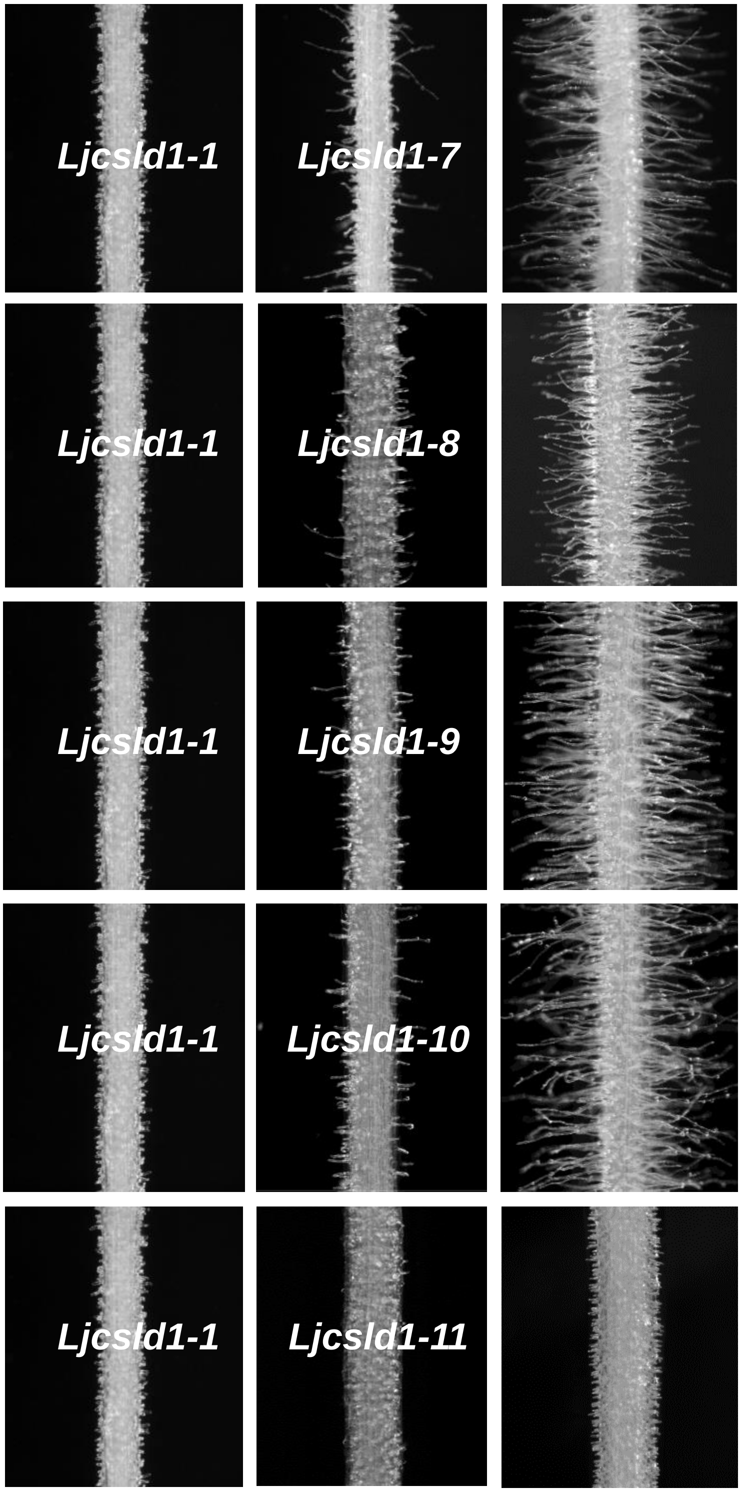

Parent 1	Parent 2	F1

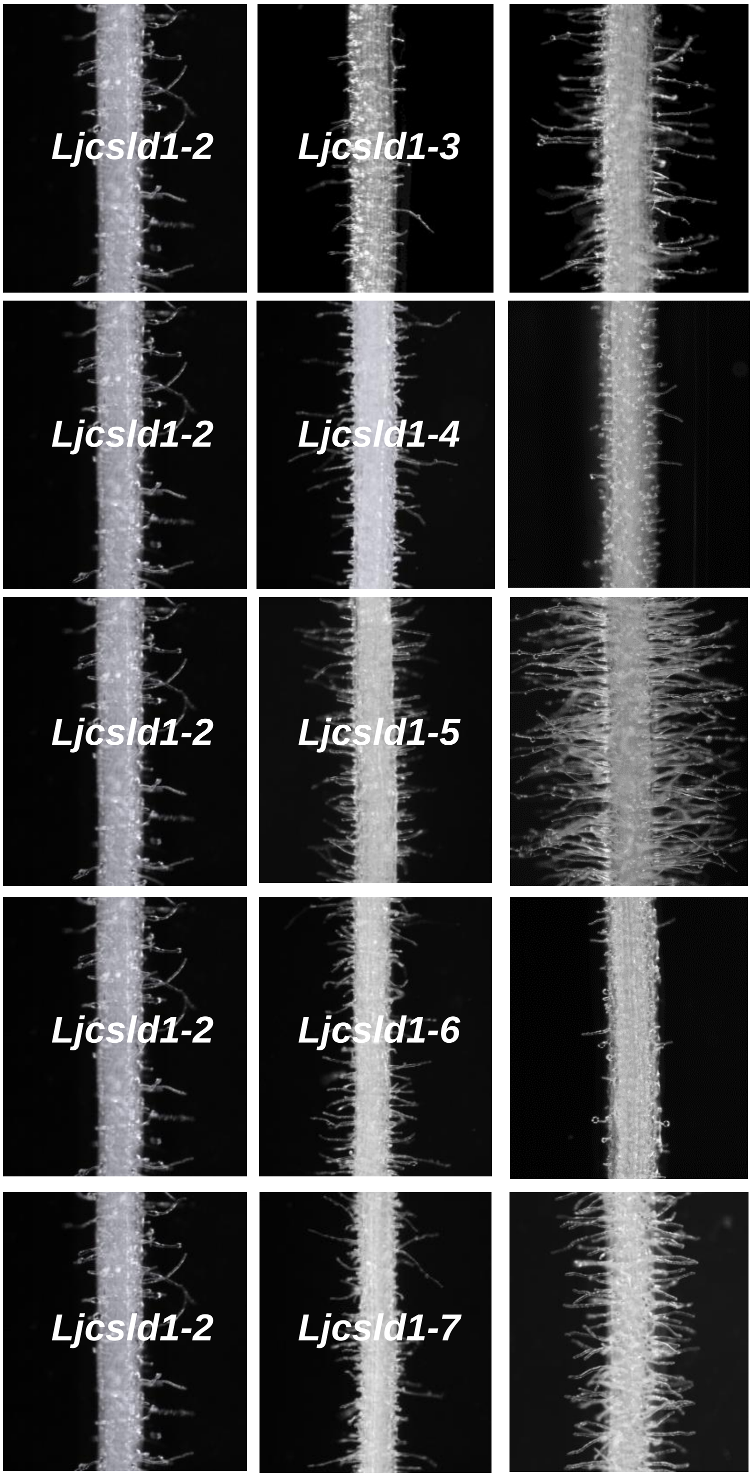

Parent 1	Parent 2	F1

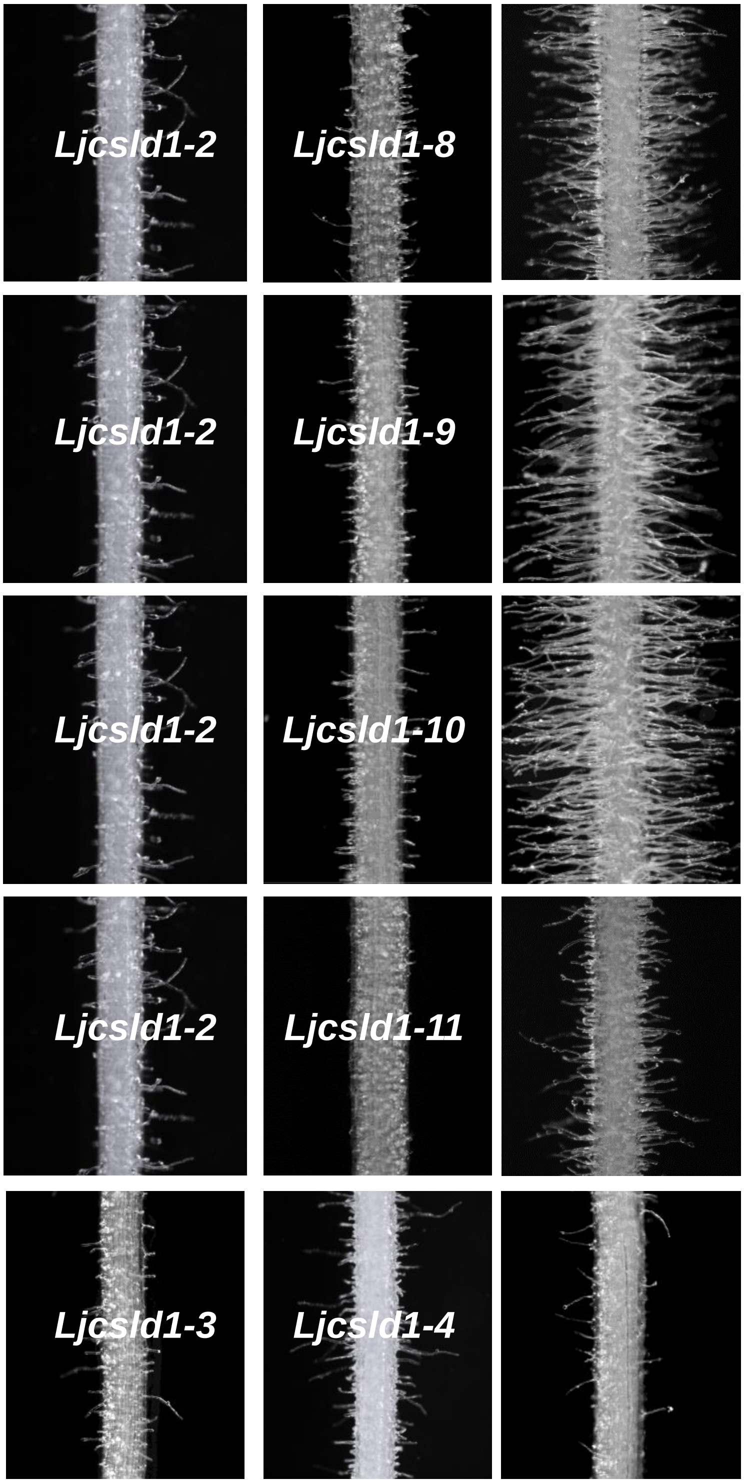

Parent 1	Parent 2	F1

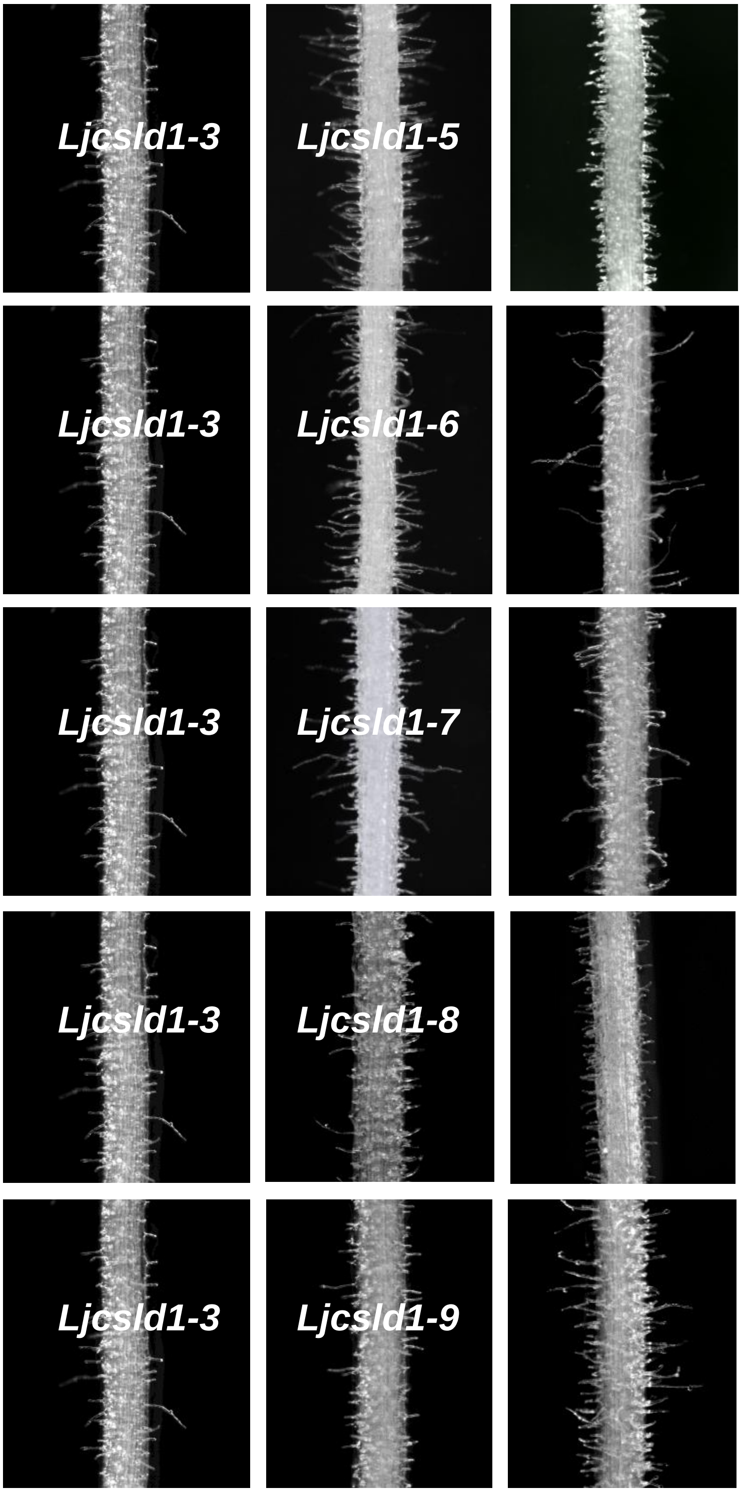

Parent 1	Parent 2	F1

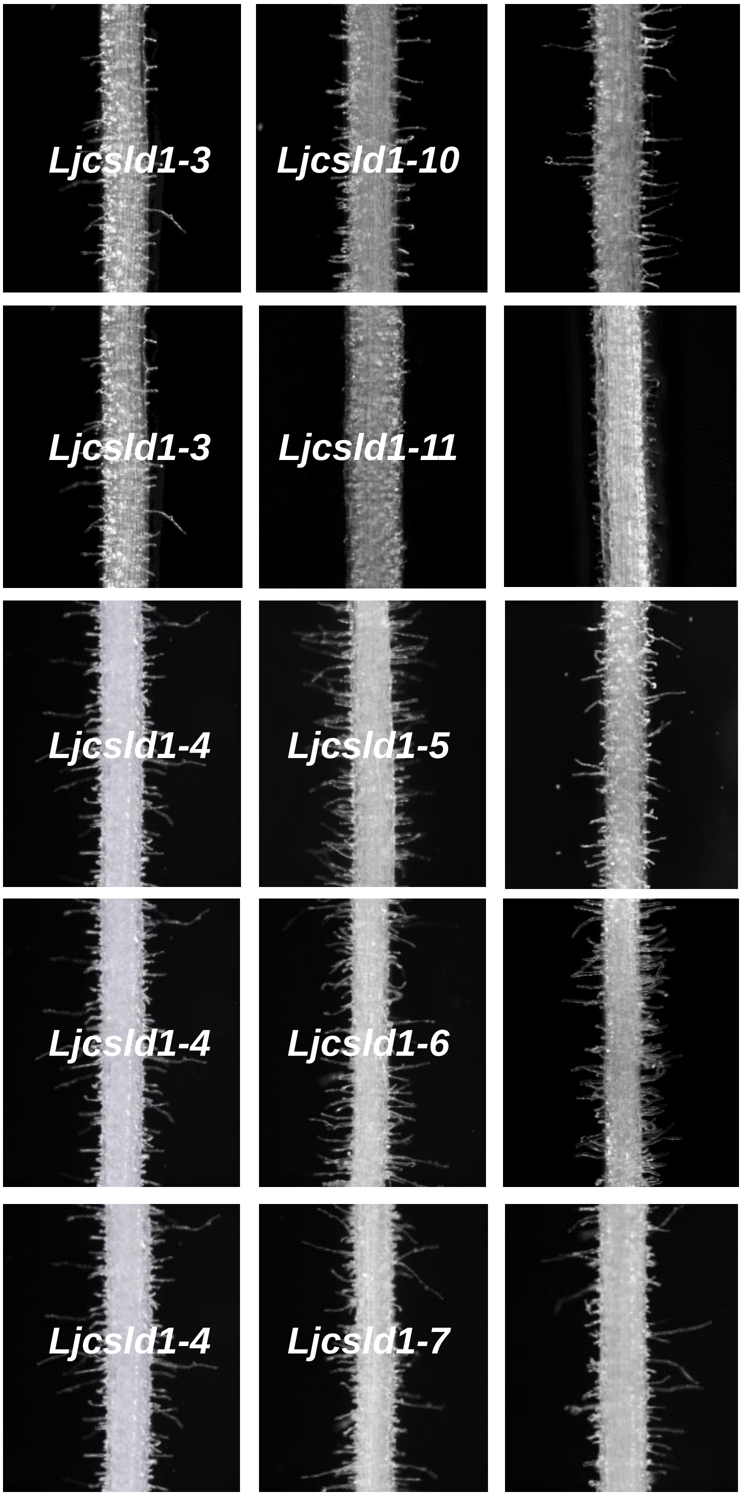

Parent 1	Parent 2	F1

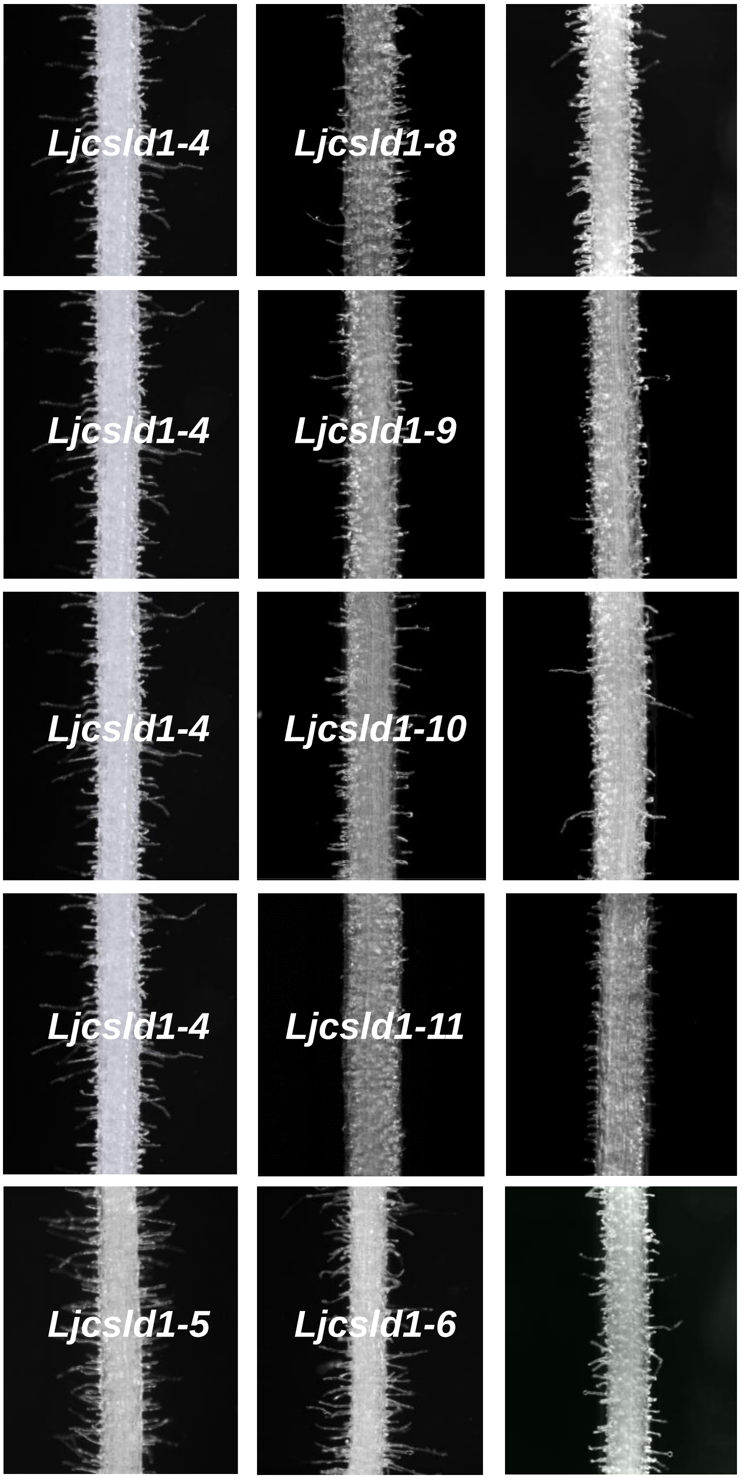

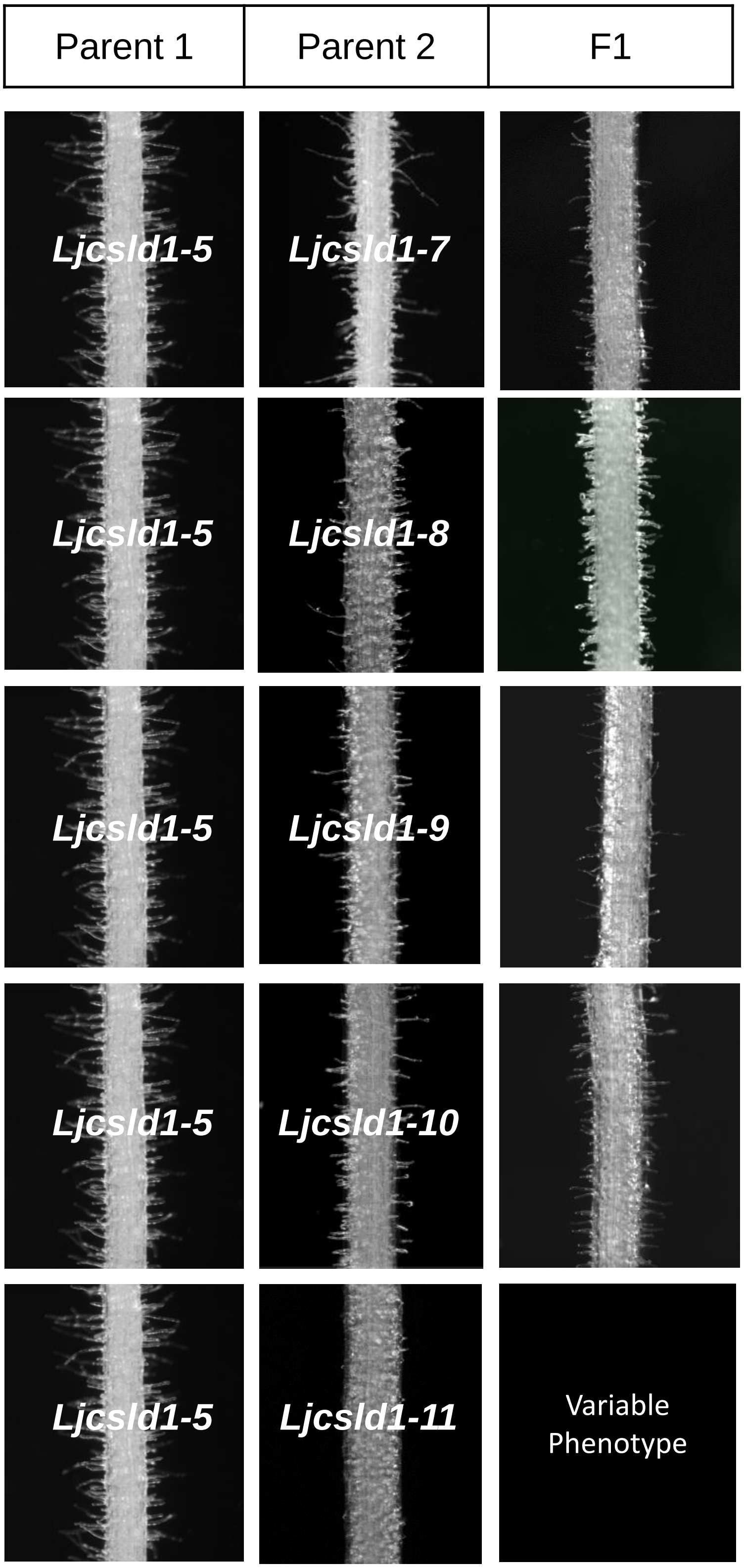
Parent 1
Parent 2
F1
Ljcsld1-5
Ljcsld1-7
Ljcsld1-5
Ljcsld1-8
Ljcsld1-5
Ljcsld1-9
Ljcsld1-5
Ljcsld1-10
Ljcsld1-5
Ljcsld1-11
Variable Phenotype

Parent 1	Parent 2	F1

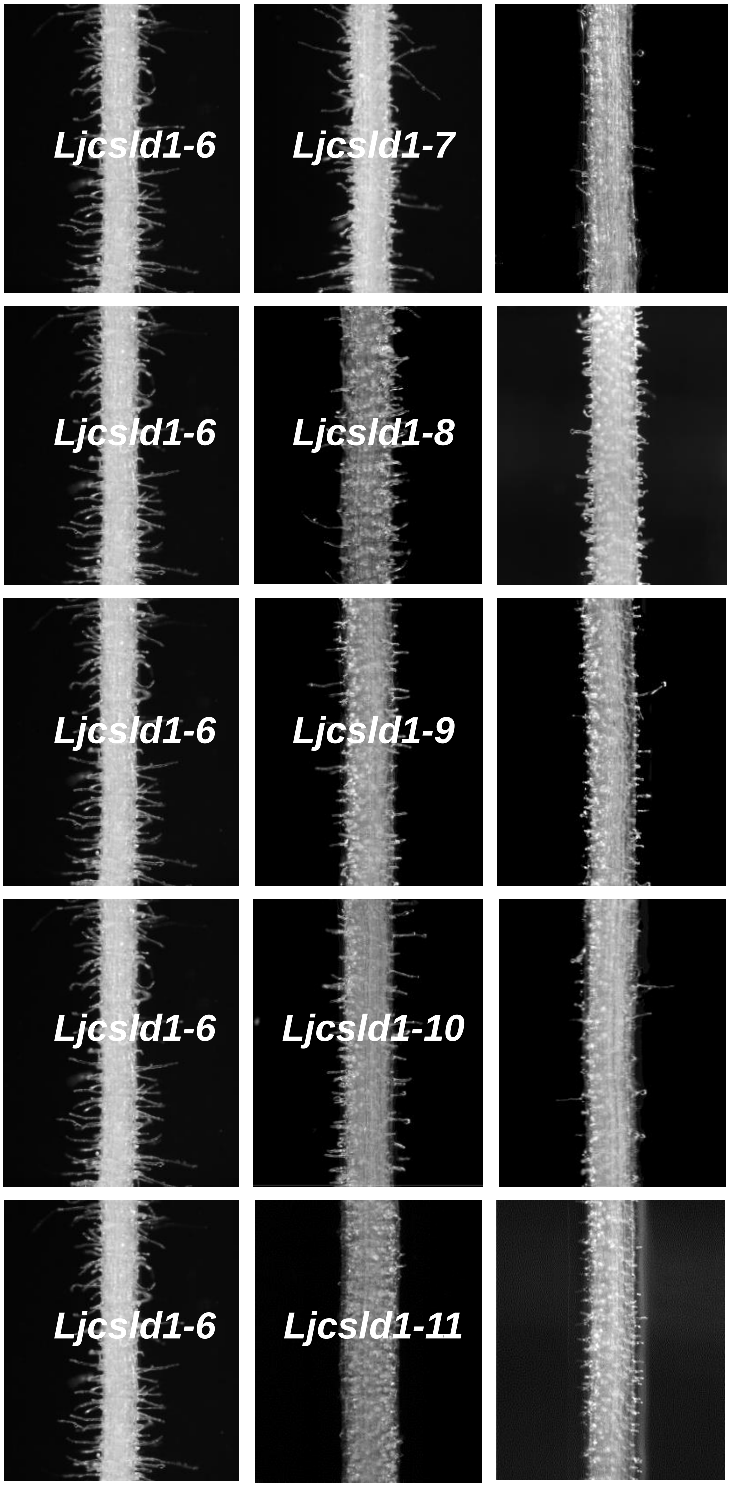

Parent 1	Parent 2	F1

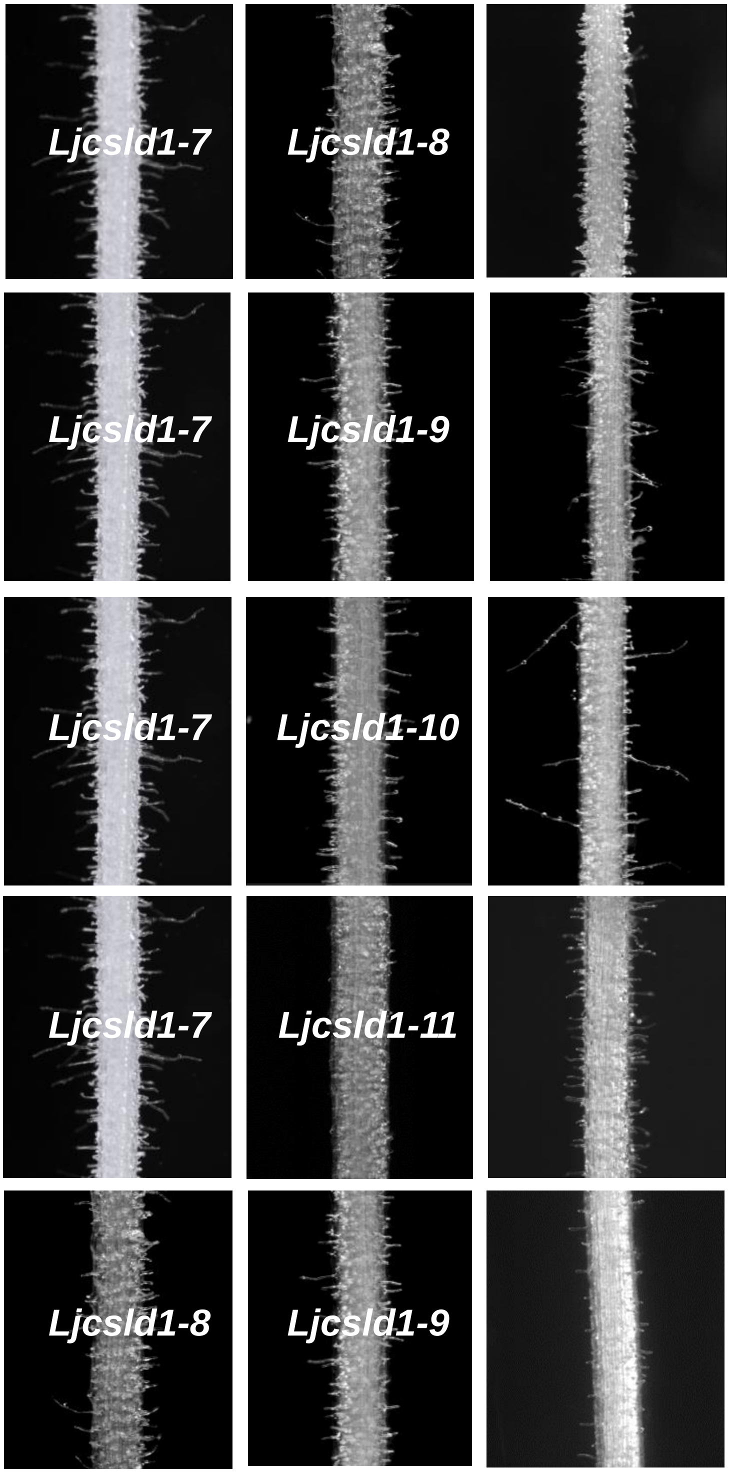

Parent 1	Parent 2	F1

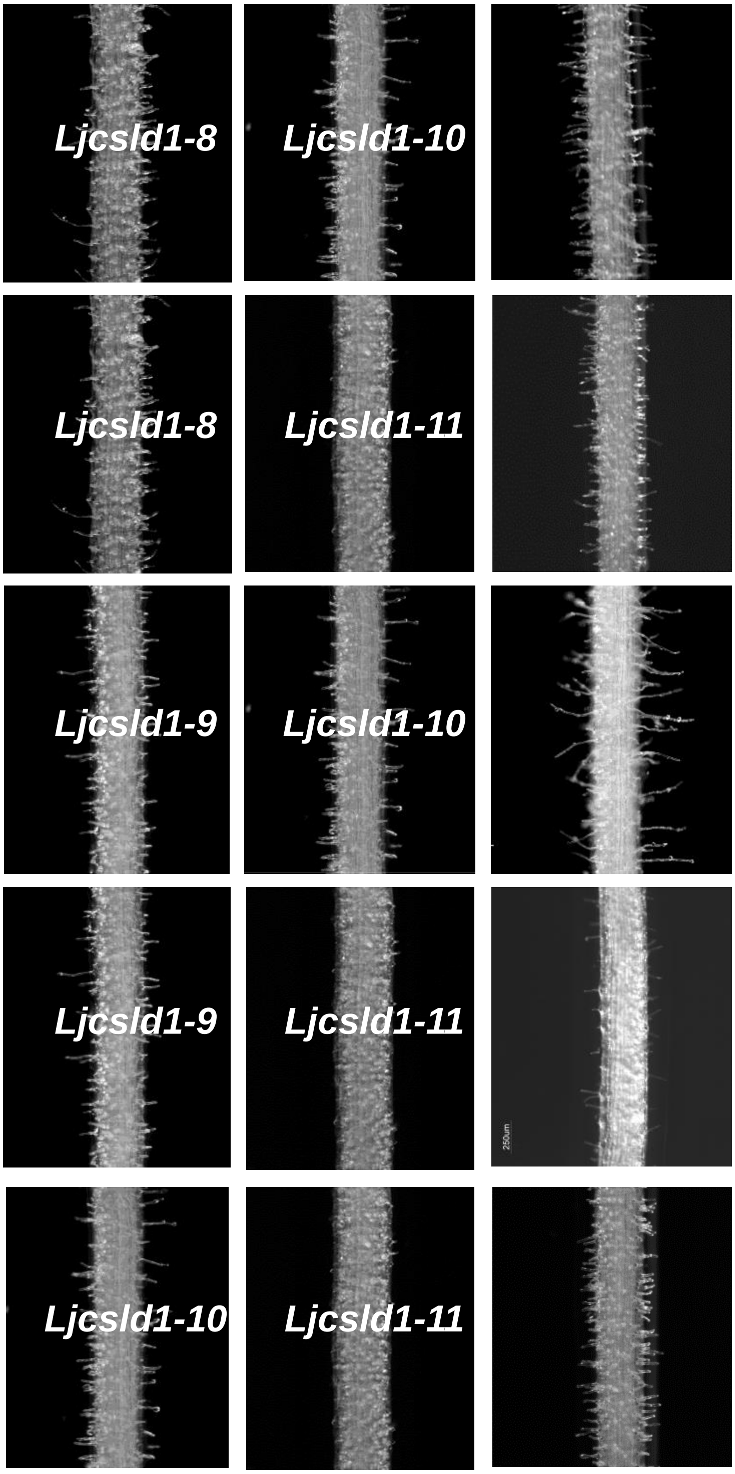

Supplement: kiab204_Supplementary_Data [file kiab204_supplementary_data.zip › pp.00250.2021-s02.pdf]
