# Supplementary material for: Intragenic complementation at the Lotus japonicus CELLULOSE SYNTHASE-LIKE D1 locus rescues root hair defects
Source: Plant Physiol. 2021 May 6;186(4):2037–50. doi: 10.1093/plphys/kiab204 (PMC8331140; doi:10.1093/plphys/kiab204)
Supplement: kiab204_Supplementary_Data [file kiab204_supplementary_data.zip › pp.00250.2021-s03.pdf]

Intragenic complementation at the *Lotus japonicus CELLULOSE SYNTHASE-LIKE D1* locus rescues root hair defects

Bogumil J. Karas[1,2], Loretta Ross[2], Mara Novero[3], Lisa Amyot[2], Arina Shrestha[1], Sayaka Inada[4], Michiharu Nakano[4], Tatsuya Sakai[5], Dario Bonetta[6], Sushei Sato[7], Jeremy Dale Murray[2,8], Paola Bonfante[3], and Krzysztof Szczyglowski[2,9]

[1]Department of Biochemistry, The University of Western Ontario, London, Ontario, N6A 5B7 Canada

[2]Agriculture and Agri-Food Canada, London Research and Development Centre, London, Ontario, N5V 4T3 Canada

[3]Department of Life Sciences and Systems Biology, University of Torino, Italy

[4]RIKEN Plant Science Center, 1-7-22 Suehiro-cho, Tsurumi-ku, Yokohama, Kanagawa 230-0045, Japan.

[5]Graduate School of Science and Technology, Niigata University, 8050 Ikarashi-nino-cho, Nishiku, Niigata 950-2181, Japan

[6]Faculty of Science, Ontario Tech University, Oshawa, Ontario, Canada

[7]Graduate School of Life Sciences, Tohoku University, 2-1-1 Katahira, Aoba-ku, Sendai, 980-8577, JAPAN

[8]National Key Laboratory of Plant Molecular Genetics, CAS-JIC Centre of Excellence for Plant and Microbial Science (CEPAMS), CAS Center for Excellence in Molecular and Plant Sciences, Institute of Plant Physiology and Ecology, Chinese Academy of Sciences, Shanghai, 200032.

[9]Department of Biology, University of Western Ontario, London, Ontario, N6A 5B7 Canada

`

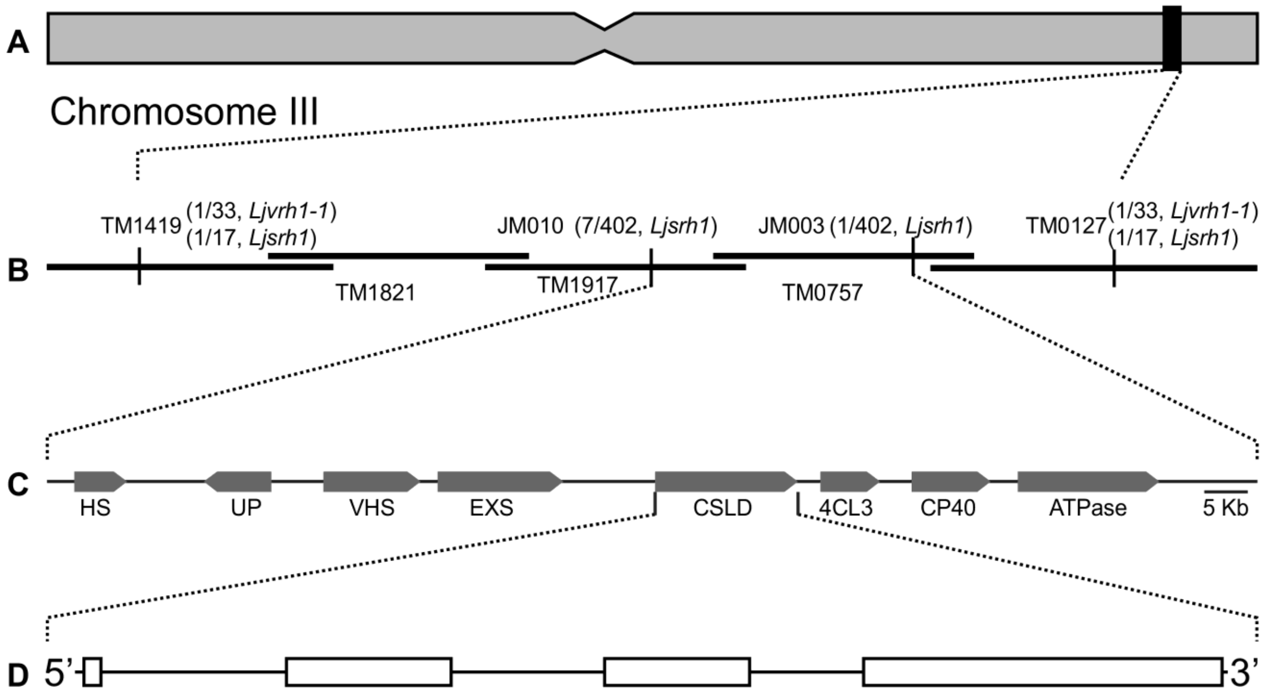

Supplemental Fig. S1. Map-based cloning of the *LjVRH1*/*LjSRH1* locus. (A) A schematic of *L. japonicus* chromosome III. (B) Overlapping TAC clones spanning the genetic interval linked to both *Ljvrh1-1* and *Ljsrh1* phenotypes; recombination frequency is given in parentheses. (C) The region linked to *Ljsrh1*, as delimited by JM010 and JM003 flanking markers, was predicted to contain eight genes. (D) Exon-intron structure of a predicted causative gene encoding a cellulose synthase-like protein.

A)

MASKPFKQSRSSLSAASDASEAQKPPLPPTVTFARRTSSGRYVNYSRDDLDSELGSELGSTDFMNYTVHLPPT**P**D
NQPMDLTVSQKVEEQYVSNSLFTGGFNSMTRAHLMDKVTESKANHPQMAGVKGSSCAVPGCDCKVMSDERGE
DILPCECDFKICRDCYIDAVKTGDGMCLG**C**KEPYKNTELDEVAVDNGRSFPLLPPNGGVSKMERRLSLMKSTKS
ALMRSQTGDFDHNRWLFETRGTYGYGNAIWPKEGNFGNGKEDGDVVDPTELMNKPWRPLTRKLKIPAAILSPY
RLIILVRFVILVLFLEWRIRHKNTDAIWLWGMSVVCELWFAFSWLLDQLPKLCPINRSTDLNVLKEKFETPSPTNP
TGKSDLPGI**D**IFVSTADPEKEPPLVTANTILSILAADYPVEKLSCYVSD**D**GGALLTFEAMAEAASFANVWVPFCRK
HDIEPRNPESYFSLKRDPYKNKVKPDFVKDRRRVKREYDEFKVRINGLPDSIRRRSDAFHAREEIKAMKLQRQNK
EDEPIEAVKIPKATWMADGTHWPGTWLNSSAEHSKGDHAGIIQVMLKPPSDEPLLGNDDDTKLIDLTDIDIRLPLL
VYVSREKRPGYDHNKKAGAMNALVRASAIMSNGPFILNLDC**D**HYIYNSKAMREGM**C**FMMDRGGDRLCYVQF**P**
QRFEGIDPSDRYANHNTVFFDVNMRALDGL**Q**GPVYVGTGCLFRRVALYGFDPPRAKEHHPGFCSCCFGRRKRIA
SHNTEENRALRMGDDDSEDEEMNLSTFPKKFGNSTFLIESIPVAEFQGRPLADHPAVKNGRPPGALTIPRELLDAA
TVAEAISVISCWYEDKTEWGQRVGWIYG**S**VTE**D**VVTGYRMHNRGWKSVYCVTKRDAFR**G**TA**P**INLTDRLH**Q**V
L**RW**ATGSVEIFFSRNNAFLASPRMKILQRIAYLNVGIYPFTSFFLIVYCFLPALSLFSGQFIVQTLSVTFLSYLLGITV
TLCILAVLEIKWSGIQLEEWWRNEQFWLIGGTSAHLAAVLQGLLKVIAGIEISFTLTSKSGGDDVDDEFADLYVFK
WTS**L**MIPPITIMMVNLIAIAVGVSRTIYSTIPQWSRLLGGVFFSFWVLTHLYPFAKGLMGRRGRTPTIVYV**W**SGLI
AITISLLWVAINPPQGANEIGGSFQFP

B)

```
AtCESA1: 23    ESDGGTKPLKNMNGQICQICGDDVGLA--ETGDVFVACNECAFPVCRPCYEYERKDGTQCCPQCKTRFRR 90
               ES        +  + G  C + G D  +    E G+  + C EC F +CR CY    K G   C  CK  ++
LjCSLD1: 115   ESKANHPQMAGVKGSSCAVPGCDCKVMSDERGEDILPC-ECDFKICRDCYIDAVKTGDGMCLGCKEPYKN 183
```

Supplemental Fig. S2. The LjCSLD1 protein contains several conserved domains. (A) The primary sequence of the LjCSLD1 protein with domains indicated by different highlights. Zinc Finger domains, $X_3CX_4CX_{15}CXC$ and $X_3CX_2CX_{11}CX_2C$, based on the consensus: $X_3CX_{2\text{-}4}CX_{12\text{-}15}CX_2C$ (Kurek et al., 2002) are indicated in red. The PPTP and eight transmembrane domains predicted by the TMHMM2.0 program (http://www.cbs.dtu.dk/services/TMHMM/) are underlined. Four conserved subdomains that characterize the processive β-glycosyl transferases are shown in blue. The first three subdomains contain D and the last contains QXXRW (bolded and highlighted in yellow). Positions of all *Ljcsld1* mutations are bolded and highlighted in green. (B) BLAST2 alignment of AtCESA1 and LjCSLD1 N-terminal regions indicating highly similar (letters) and similar (+) sequences. The cysteines of the CESA1 zinc fingers are indicated in bold red. Other LjCSLD1 cysteines are indicated in bold. The cysteine residue mutated in *Ljcsld1-2* is highlighted in grey.

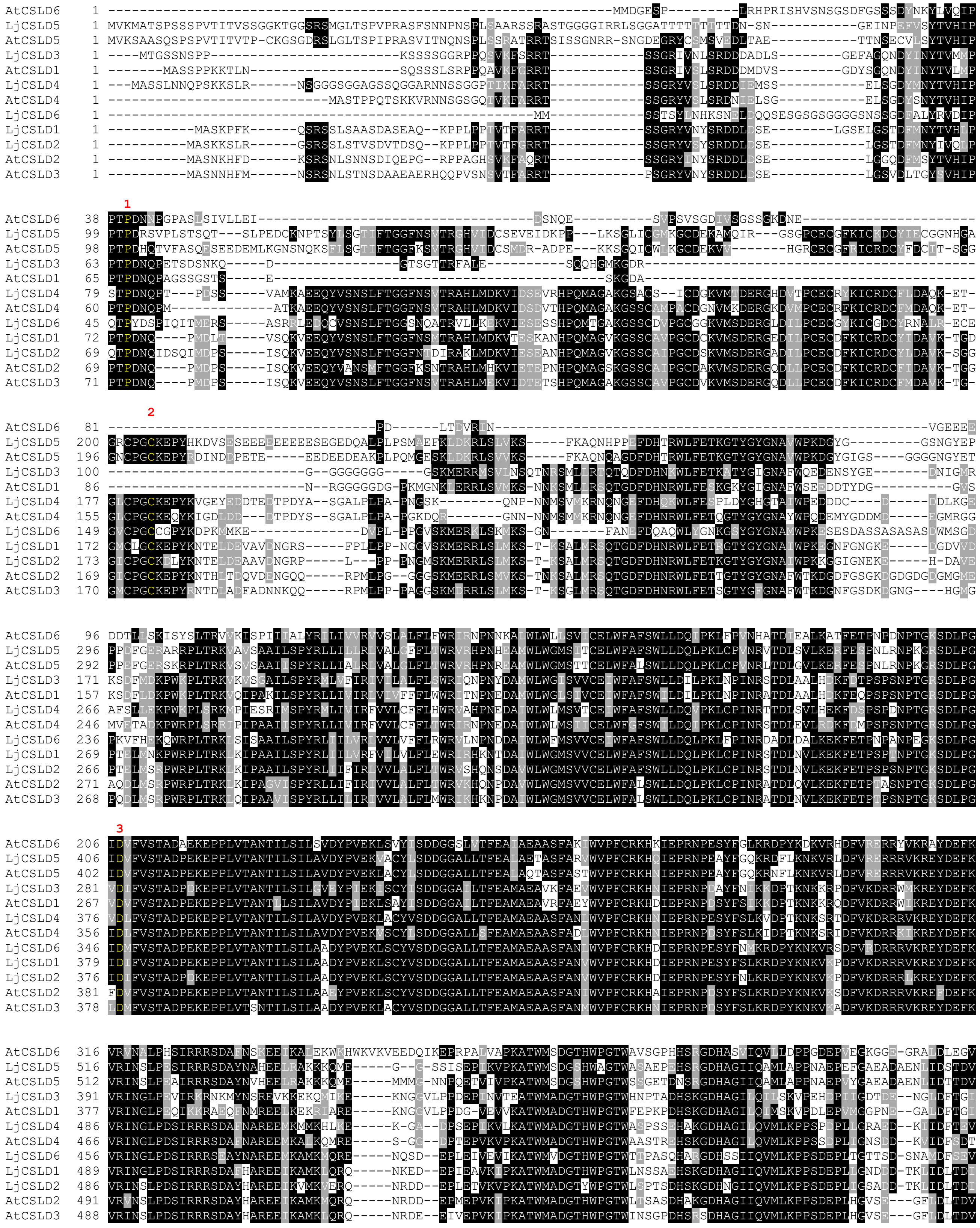

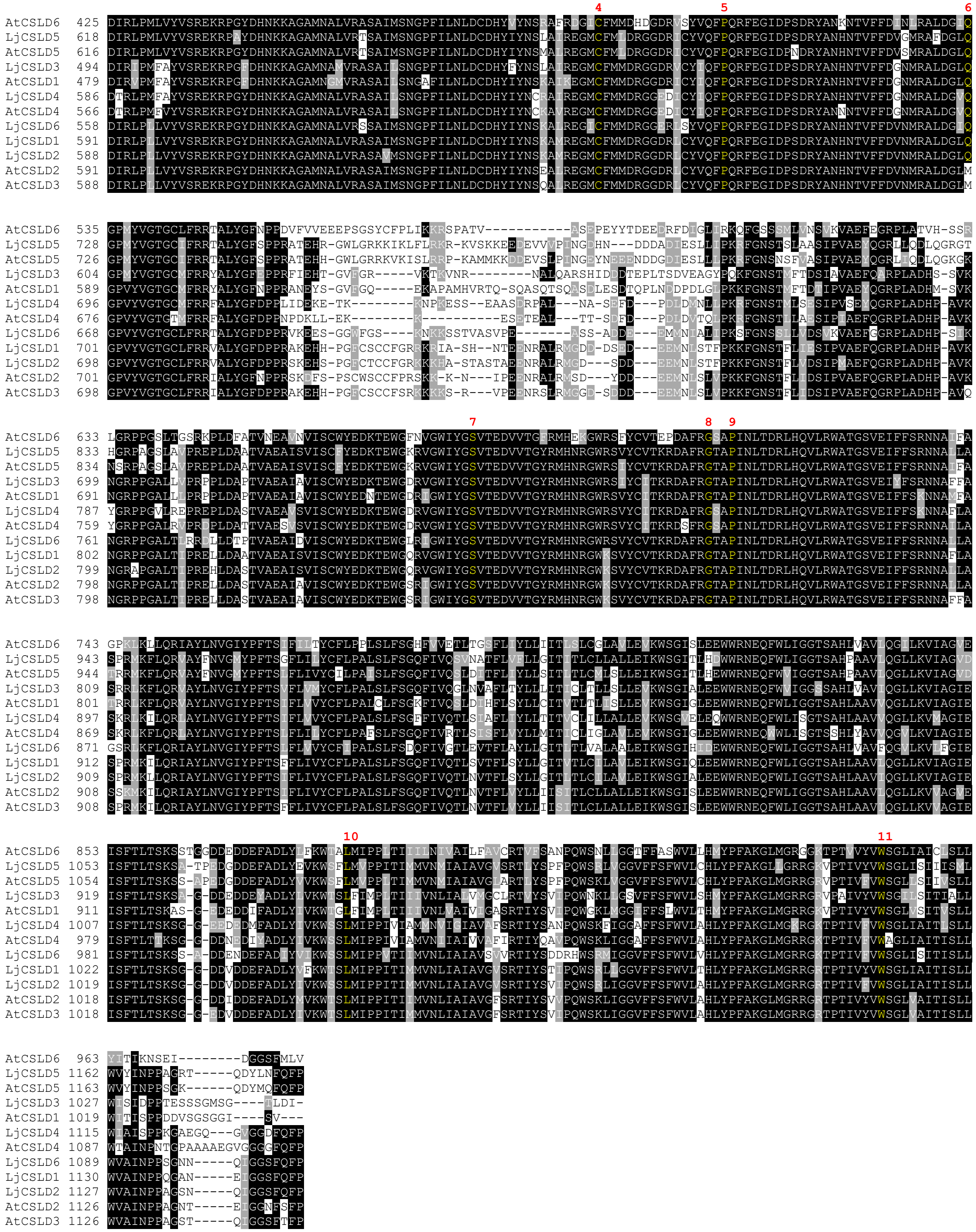

Supplemental Fig. S3. Alignment of predicted CSLD protein sequences of *L. japonicus* and *A. thaliana*. The predicted CSLD protein sequences were aligned with Clustal Omega using the default

settings and the final output was generated using the BoxShade Server version 3.21. A threshold of ≥ 50% conservation was used. Black shading specifies identical residues, whereas grey indicates the presence of conservative substitutions. The positions of all mutations are highlighted by numbers (1 to 11) and yellow text.

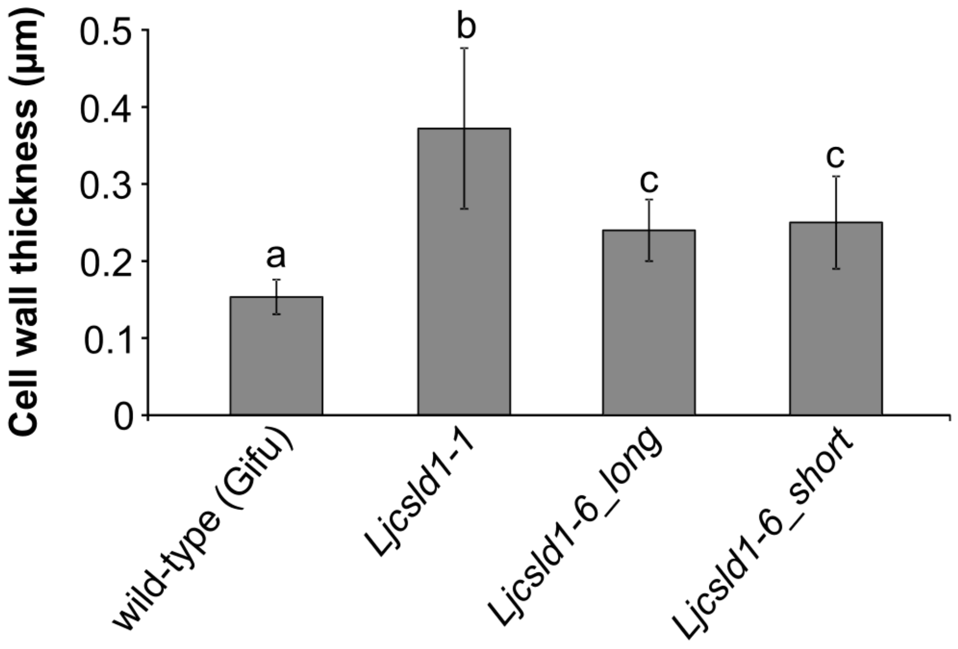

Supplemental Fig. S4. *Ljcsld1-1* and *Ljsld1-6* significantly increase cell wall thickness of mature root hairs. Transmission electron microscopic (TEM) images of root hair longitudinal sections for each genotype and the corresponding root hair type were used in this analysis. Cell wall thickness of the middle part of a given root hair was measured at four different points and the mean value was derived for each TEM image. The graph represents mean values ± 95% CI for five to ten independent root hairs per given genotype. Statistical grouping is indicated by the same lower-case letter ($P<0.05$, Kruskal Wallis test).

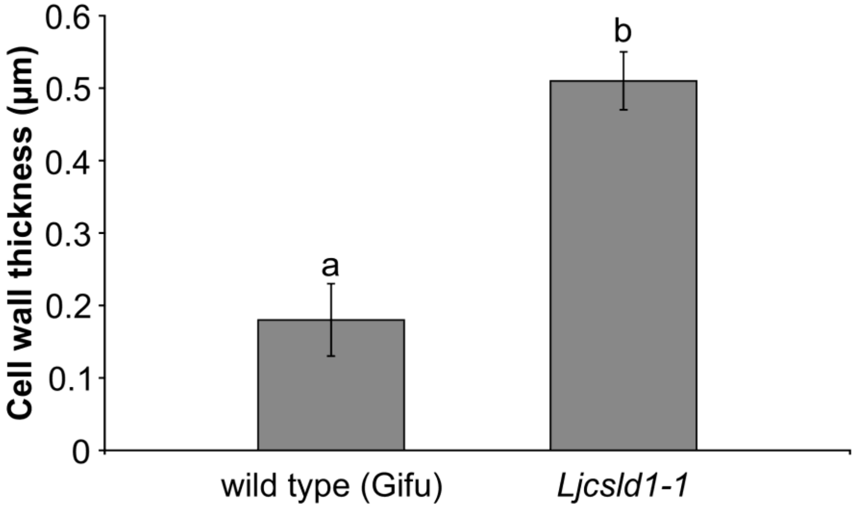

Supplemental Fig. S5. *Ljcsld1-1* significantly increases the root hair cell wall thickness. Measurements were taken from regions where the young, growing wild-type root hairs were of similar lengths as those formed on *Ljcsld1-1* mutant roots. Measurements were performed as described in the legend to Supplemental Figure S4. 12 and 17 independent root hairs were measured for wild-type (Gifu) and *Ljcsld1-1*, respectively. Statistical grouping is indicated by the same lower case letter ($P<0.05$, Kruskal Wallis test)

Supplemental Table S1. List of *L. japonicus* mutant lines and corresponding *Ljcsld1* alleles. Positions of mutations (where adenine of the ATG initiation codon is counted as 1) and their respective impacts on the predicted LjCSLD1 protein sequence (where a predicted initiation methionine is counted as 1) are given. Note that bold letters denote the ecotype MG20 background while lines in grey text represent *L. japonicus* Gifu.

Mutant name	Allele name	Nucleotide substitution	Amino acid change
Ljsrh1	*Ljcsld1-1*	C_{220} → T	P_{74} → S
Ljvrh1-3	*Ljcsld1-2*	G_{530} → A	C_{177} → Y
**210-226a**	***Ljcsld1-3***	**G_{1933} → A**	**D_{380} → N**
S36-1	*Ljcsld1-4*	G_{3354} → A	C_{653} → Y
**212-010**	***Ljcsld1-5***	**C_{3401} → T**	**P_{669} → S**
Ljvrh1-1	*Ljcsld1-6*	C_{3494} → T	Q_{700} → Stop
Ljvrh1-2	*Ljcsld1-7*	C_{3939} → T	S_{848} → L
**212-229**	***Ljcsld1-8***	**G_{4029} → A**	**G_{878} → D**
**01-0071**	***Ljcsld1-9***	**C_{4038} → T**	**P_{881} → L**
**212-164**	***Ljcsld1-10***	**C_{4544} → T**	**L_{1050} → F**
**212-447**	***Ljcsld1-11***	**G_{4750} → A**	**W_{1118} → Stop**

Supplemental Table S2. Identity and similarity values for comparison of full-length *L. japonicus* and Arabidopsis CSLD proteins. The sequence identity and similarity were generated using the blastp algorithm at NCBI.

**AtCSLD vs. LjCSLD protein identity/similarity [%]**						
Gene Name	LjCSLD1	LjCSLD2	LjCSLD3	LjCSLD4	LjCSLD5	LjCSLD6
AtCSLD1	71/84	70/83	76/86	71/82	64/78	67/80
AtCSLD2	90/90	83/90	70/83	70/81	66/77	70/81
AtCSLD3	85/92	83/90	71/83	69/80	66/77	70/80
AtCSLD4	73/83	71/92	69/83	80/90	62/75	67/79
AtCSLD5	65/77	64/77	66/79	61/73	79/87	63/74
AtCSLD6	68/80	66/80	63/77	66/80	65/79	69/82

Supplemental Table S3. Additional information about LjCSLD1 genomic, mRNA and protein sequences.

**Sequence**	**Length (bp)**
Genomic sequence	6197
mRNA sequence	3805
5’UTR	152
3’UTR	203
ORF	3450
**Gene Structure**	**Start-End (bp)**
Intron 1	120-1097
Exon 1	1-119, 1098-2014
Intron 2	2015-2809
Exon 2	2810-3617
Intron 3	3618-4218
Exon 3	4219-6179
**LjCSLD1 Protein**	**Predicted size**
Length	1149aa
Mass (using Lasergene Protean)	129.261kDa

Supplemental Table S4. Analyses of cell wall composition in *L. japonicus* wild type and *Ljcsld1* mutant roots. All values are given as nmol/mg dry root weight ± 95% CI. (A) Measurements for the cellulose fraction and neutral sugars from the non-cellulosic fraction are given. (B) Comparison of pectin and hemicellulose fractions of the root cell walls. Values that are statistically different from the wild-type control were defined by using the Student's *t*-test (P<0.05) and are highlighted. *cellulose measured as glucose equivalents; **monosaccharides quantified as alditol acetates; ***pectin expressed as galacturonic acid equivalents.

A

Allele	Wild type	*Ljcsld1-1*	*Ljcsld1-2*	*Ljcsld1-4*	*Ljcsld1-6*	*Ljcsld1-7*
Cellulose*	986.9 ± 30.5	1083.4 ± 60.7	840.2 ± 15.2	814.0 ± 48.1	839.9 ± 29.5	869.1 ± 39.4
Neutral Sugars from Non-Cellulosic fraction**						
Rhamnose	53.8 ± 4.6	55.2 ± 5.1	53.2 ± 3.9	54.7 ± 3.4	60.6 ± 10.1	47.1 ± 6.0
Fucose	30.1 ± 1.4	30.8 ± 1.4	24.4 ± 2.1	23.5 ± 1.8	24.8 ± 2.3	24.9 ± 1.3
Arabinose	408.4 ± 15.9	418.8 ± 10.7	359.8 ± 24.5	345.8 ± 13.8	389.5 ± 37.5	353.9 ± 4.6
Xylose	177.1 ± 5.9	172.5 ± 4.1	142.0 ± 7.0	138.4 ± 8.0	146.9 ± 11.9	139.4 ± 4.2
Mannose	39.5 ± 5.7	24.3 ± 3.1	26.0 ± 2.8	33.1 ± 6.2	24.0 ± 0.8	23.7 ± 1.9
Galactose	224.2 ± 6.7	195.4 ± 10.2	164.7 ± 7.3	157.7 ± 5.4	172.7 ± 23.2	156.6 ± 2.3
Glucose	104.4 ± 10.5	92.2 ± 14.0	121.8 ± 10.7	128.4 ± 23.1	122.3 ± 26.4	147.3 ± 19.0

B

Allele	Wild type	*Ljcsld1-1*	*Ljcsld1-2*	*Ljcsld1-4*	*Ljcsld1-6*	*Ljcsld1-7*
Pectin***	386.9 ± 17.1	408.8 ± 18.9	412.5 ± 17.2	382.7 ± 38.2	419.4 ± 13.1	378.6 ± 41.3
Neutral Sugars from Hemicellulose fraction**						
Rhamnose	21.6 ± 4.0	21.3 ± 4.3	21.9 ± 4.4	16.0 ± 2.7	25.6 ± 2.9	17.2 ± 2.0
Fucose	16.5 ± 1.7	15.8 ± 2.1	17.8 ± 2.3	15.2 ± 1.4	14.6 ± 0.5	16.5 ± 2.9
Arabinose	129.9 ± 11.5	143.9 ± 17.0	167.3 ± 13.0	132.5 ± 8.9	145.1 ± 9.1	137.2 ± 23.9
Xylose	95.1 ± 9.2	103.8 ± 10.4	104.9 ± 5.2	78.7 ± 5.5	108.3 ± 5.8	81.1 ± 13.6
Mannose	22.9 ± 2.8	14.4 ± 2.6	17.5 ± 2.4	14.7 ± 1.4	13.8 ± 0.4	15.8 ± 3.8
Galactose	65.7 ± 6.0	64.1 ± 7.5	68.4 ± 3.8	53.1 ± 2.6	61.6 ± 0.5	53.5 ± 10.3
Glucose	156.5 ± 34.0	146.3 ± 36.4	164.4 ± 37.4	109.7 ± 13.7	196.3 ± 8.2	113.8 ± 4.0

Supplemental Table S5. List of primers used in this study. All sequences are in 5' to 3' orientation.

**Experiment**	**Primer name**	**Primer sequence**
Map-based cloning		
	JM003_F	TCGGAGACAGAAGGCATCTT
	JM003_R	GCTTGTCTGGGAAGCTGTTC
	JM010_F	ACTATACGTCGCACCAAACG
	JM010_R	GCCATGACTGGAGCAGAAAC
Amplification of LjCSLD1 cDNA		
	CSLD1_cDNAF	TGACAGTGAGCTGGGAAGTG
	CSLD1_cDNAR	AGCACCCAAAAGCTGAAGAA
Rapid Amplification of cDNA Ends (RACE)		
	3'outer race	GTCGATGACGAGTTTGCTGA
	3'inner race	CAGCACCATACCTCAGTGGA
	5'outer race	GCTGTTGAATCCTCCGGTAA
	5'inner race	TTGGTTATCAGGGGTTGGTG
Evaluation of LjCSLD1 in L. japonicus tissues		
	LjCSLD1expF	CCGTTTGCTAGGTGGTGTTT
	LjCSLD1expR	TCACCATCAAACGTTCCTGA
	Ubi-F	TTCACCTTGTGCTCCGTCTTC
	Ubi-R	AACAACAGAACACACAGACAATCC
Construction of LjCSLD1p::GUS plasmid		
	LjCSLD1prmt_F	TCTAGATCTAGATTTTGTTTTGGTCAACAGCAG
	LjCSLD1prmt_R	CCCGGGCCCGGGTACACTGGCACACGGAGAGA
Construction of LjCSLD1p::LjCSLD1 plasmid		
	LjCSLD1compF	GGGGACAAGTTTGTACAAAAAAGCAGGCTTTGGTCCACAAACAGCTGAA
	LjCSLD1compR	GGGGACCACTTTGTACAAGAAAGCTGGGTTCACCATCAAACGTTCCTGA
Construction of 35Sp::LjCSLD1, AtCSLA9, AtCSLD2, AtCSLD3 plasmids		
	Ljcsld1_35S_F	GGGGACAAGTTTGTACAAAAAAGCAGGCTTCCGTGTGCCAGTGTATGTT
	Ljcsld1_35S_R	GGGGACCACTTTGTACAAGAAAGCTGGGTGAGACAACAAAAAGCCTTGGA
	Atcsla9_F	GGGGACAAGTTTGTACAAAAAAGCAGGCTTCCTTTTTCCCGACAATCTG
	Atcsla9_R	GGGGACCACTTTGTACAAGAAAGCTGGGTTGCCTTCAAGGAATCTGAAAA
	Atcsld2_F	GGGGACAAGTTTGTACAAAAAAGCAGGCTAAAGATGTGTGGGCTTTTCG
	Atcsld2_R	GGGGACCACTTTGTACAAGAAAGCTGGGTTCCACCCAATCTTGTTCCAT
	Atcsld3_F	GGGGACAAGTTTGTACAAAAAAGCAGGCTTGTCCGAAAACCTCAACACA
	Atcsld3_R	GGGGACCACTTTGTACAAGAAAGCTGGGTCCCGTTTTGTTTCCTTTTCC

References

Kurek I, Kawagoe Y, Jacob-Wilk D, Doblin M, Delmer D (2002) Dimerization of cotton fiber cellulose synthase catalytic subunits occurs via oxidation of the zinc-binding domains. Proc Natl Acad Sci U S A **99**: 11109–14
